# Decoding Diffuse Midline Gliomas: A Comprehensive Review of Pathogenesis, Diagnosis and Treatment

**DOI:** 10.3390/cancers15194869

**Published:** 2023-10-06

**Authors:** Sarah Al Sharie, Dima Abu Laban, Maysa Al-Hussaini

**Affiliations:** 1 Faculty of Medicine, Yarmouk University, Irbid 21163, Jordan; sarahalsharie2000@gmail.com; 2 Department of Radiology, King Hussein Cancer Center, Amman 11941, Jordan; da.11945@khcc.jo; 3 Department of Pathology and Laboratory Medicine, King Hussein Cancer Center, Amman 11941, Jordan

**Keywords:** diffuse midline glioma, H3 K27M, H3 K27me3, prognosis, brainstem, thalamus, spinal cord

## Abstract

**Simple Summary:**

Diffuse midline glioma (DMG) is a highly aggressive brain tumor primarily affecting children and young adults. It grows diffusely in midline structures. Unfortunately, DMG has a very poor prognosis, rendering traditional treatment such as radiation therapy and chemotherapy limited in controlling tumor growth. Ongoing research aims to understand the tumor’s biology and develop new therapies. Genomic profiling has identified specific mutations, such as H3F3A and HIST1H3B gene alterations, which may offer potential targets for future treatment. However, managing DMGs remains challenging, and more effective therapies are desperately needed to improve outcomes in affected individuals.

**Abstract:**

Diffuse midline gliomas (DMGs) are a group of aggressive CNS tumors, primarily affecting children and young adults, which have historically been associated with dismal outcomes. As the name implies, they arise in midline structures in the CNS, primarily in the thalamus, brainstem, and spinal cord. In more recent years, significant advances have been made in our understanding of DMGs, including molecular features, with the identification of potential therapeutic targets. We aim to provide an overview of the most recent updates in the field of DMGs, including classification, molecular subtypes, diagnostic techniques, and emerging therapeutic strategies including a review of the ongoing clinical trials, thus providing the treating multidisciplinary team with a comprehensive understanding of the current landscape and potential therapeutic strategies for this devastating group of tumors.

## 1. Introduction

Diffuse midline gliomas (DMGs) are a rare type of CNS tumors that arise in midline structures such as the thalamus, brainstem, and spinal cord [1,2]. They are classified as high-grade gliomas, an aggressive malignant tumor often associated with poor prognosis [1]. These tumors were previously referred to as diffuse intrinsic pontine gliomas (DIPGs) as they predominated in the brainstem, particularly in the pons. More recently, the term DMG has been adopted to encompass similar tumors that occur in other midline locations in the brain and spinal cord [3,4].

As the name implies, DMGs are characterized by diffuse infiltrative growth patterns [5]. They are composed of glial cells, mostly astrocytes, which are the supportive cells that make up the central nervous system that have undergone genetic mutations, resulting in malignant transformation [6,7,8].

The hallmark genetic alteration in DMGs is a point mutation in the histone H3 gene, specifically H3F3A or HIST1H3B/C, resulting in the substitution of a lysine (K) with a methionine (M) at the 28th amino acid position (K27M) [9,10]. This mutation occurs in the tail domain of histone H3, which is responsible for regulating gene expression by modulating chromatin structure [1,11]. The H3 K27M mutation leads to a global reduction in tri-methylation of histone H3 at lysine 27 (H3 K27me3), a modification associated with the loss of gene repression and instead promotes an increase in mono-methylation of H3 K27 (H3 K27me1), a mark linked to active gene transcription. This epigenetic dysregulation disrupts normal gene expression patterns and drives tumor growth and progression [12,13,14].

DMGs predominantly occur in children and young adults, with a peak incidence in children between 5 and 10 years of age [15,16]. The exact cause of DMGs is unknown, and there are currently no known risk factors or preventive measures correlated with these tumors [17]. DMGs are typically diagnosed based on clinical presentation, neuroimaging studies such as magnetic resonance imaging (MRI), and sometimes confirmed through biopsy or post-mortem examination [5].

The prognosis for DMGs remains poor, with a median survival of less than one year from the time of diagnosis [18]. DMGs are highly aggressive and tend to infiltrate surrounding brain tissue, making any attempt at surgical removal nearly impossible [19,20]. Additionally, DMGs are resistant to traditional chemotherapy and radiation therapy, which are standard treatments for other types of brain tumors [21]. The location of DMGs in critical areas of the brain and the integrity of the blood brain barrier further limits the ability to deliver targeted therapy effectively [5,22].

Given the challenges in treating DMGs, there has been growing interest in research and clinical trials to explore novel treatment approaches. Advances in molecular profiling and genetic sequencing have shed light on the unique genetic characteristics of DMGs, providing potential targets for new therapies. Targeted therapies, immunotherapies, and other novel treatment strategies are being investigated in clinical trials with the goal of improving outcomes for patients with DMGs [23]. In this article, we aim to provide an up-to-date, comprehensive review of the pathogenesis, diagnosis, and treatment methods of DMGs.

## 2. The WHO Classification

Over the years, the World Health Organization (WHO) has updated its classification of DMGs to better understand their molecular features, prognosis, and guide treatment decisions. The 2016 WHO Classification of Tumors of the Central Nervous System introduced a significant change in the classification of DMGs [3].

Prior to 2016, DMGs were classified based on their location and were referred to as gliomas located in the brainstem or thalamus. However, the 2016 update recognized that DMGs share common molecular features despite their diverse locations and introduced a new diagnostic category called “Diffuse Midline Glioma, H3 K27M-mutant” which replaced the previously used terms like “brainstem glioma” or “thalamic glioma” [3]. The 2016 classification recognized the presence of a specific mutation in the H3 histone gene, specifically H3F3A or HIST1H3B, which resulted in a substitution of lysine by methionine at position 28 (K27M). This mutation was found to be highly prevalent in DMGs and was considered a defining molecular feature of this tumor entity [24]. The 2016 classification also identified other molecular alterations associated with DMGs, such as Tumor Protein 53 (TP53), Alpha-Thalassemia/mental Retardation, and X-linked (ATRX) mutations, which further contributed to the understanding of the molecular landscape of DMGs [24].

However, the 2016 WHO classification had some limitations as it did not fully capture the heterogeneity and complexity of DMGs. There were still some cases of DMGs that lacked the characteristic H3 K27M mutation or had other unique molecular features. To address these limitations, the WHO updated the classification in 2021 with further refinements for DMGs [25,26].

The 2021 WHO Classification of Tumors of the Central Nervous System introduced several changes to the classification of DMGs. One of the major changes was the nomenclature of DMG, “H3 K27M mutant”, was replaced by “DMG, H3 K27-altered” to indicate the fact that this entity can be defined by alterations other than the previously known H3 K27 mutations (for example, EZHIP protein overexpression) [25,26]. Moreover, they introduced a new category called “Diffuse Midline Glioma, H3 K27M-mutant, Not Otherwise Specified (NOS)”, which included DMGs that have the characteristic H3 K27M mutation but lack specific histologic or molecular features to classify them into other defined entities [25,26]. This category was useful for cases that did not fit into other well-defined molecular subtypes of DMGs, allowing for a more comprehensive and accurate classification of DMGs.

Another important addition in the 2021 classification was the recognition of other molecular alterations in DMGs that can help in their subclassification. For example, DMGs with mutations in the Activin A receptor 1 (ACVR1) gene, known as diffuse intrinsic pontine glioma (DIPG)-like ACVR1-mutant gliomas, were identified as a distinct molecular subgroup within DMGs [25,26]. These tumors have specific histologic and molecular features that differentiate them from other DMGs, and their recognition allowed for more precise classification and potential targeted therapeutic approaches [27,28].

Furthermore, the 2021 classification also recognized other molecular alterations in DMGs, such as alterations in the MYB and MYBL1 genes, which were identified in a subset of DMGs and were associated with a better prognosis. These molecular alterations further contributed to the understanding of the molecular heterogeneity of DMGs and may guide personalized treatment strategies [25].

The 2021 WHO classification also highlighted the importance of incorporating histologic features in the classification of DMGs. While molecular features are crucial, histologic characteristics such as mitotic activity, vascular proliferation, and necrosis can also provide valuable information for the diagnosis and prognosis of DMGs. Histologic features are typically assessed in combination with molecular alterations to obtain a comprehensive classification of DMGs [25].

The updated WHO classification also underscored the importance of obtaining a multidisciplinary approach in the diagnosis and management of DMGs. It highlighted the need for integrating radiologic, histologic, and molecular findings to achieve a more accurate classification and guide treatment decisions. Additionally, the updated classification encourages the use of molecular testing, such as DNA sequencing and surrogate immunohistochemistry when available, to identify the specific molecular alterations associated with DMGs, as this information has important implications for prognosis and treatment selection [25].

## 3. Biological Aspects of DMGs

**Figure 1:**
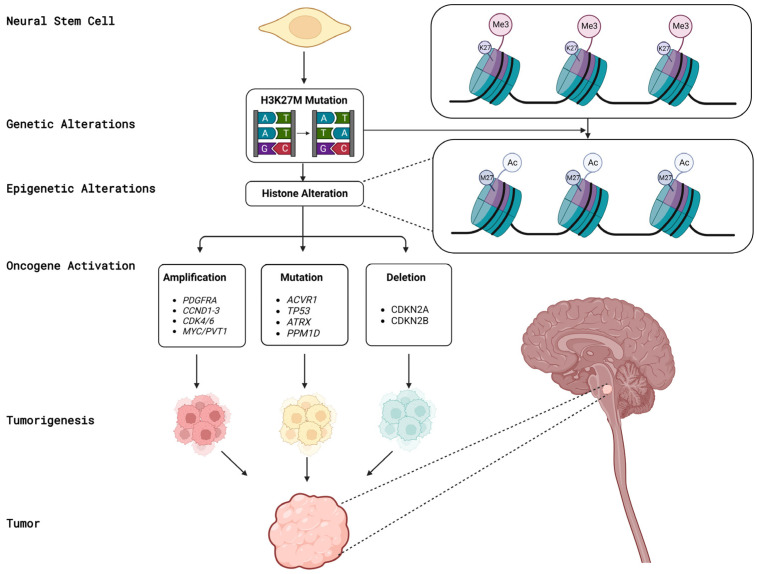
Schematic summary of the genes implicated in the pathogenesis of DMG (Adopted with modifications from reference [5]).

Several genetic alterations have been identified in DMGs that may contribute to tumor development and progression. These alterations include mutations in histone genes, such as H3 K27M, as well as alterations in various signaling pathways, such as the receptor tyrosine kinase platelet-derived growth factor receptor Alfa (PDGFRA), ACVR1, the Mitogen-Activated Protein Kinase (MAPK) pathway, and the H3-wildtype DMGs. In addition, alterations in tumor suppressor genes, such as TP53, Cyclin-Dependent Kinase inhibitor 2A/2B (CDKN2A/CDKN2B), and MHC class I polypeptide-related sequence A (MICA), have also been implicated in DMG pathogenesis [29]. Figure 1 provides a summary of the genes implicated in the pathogenesis of DMG.

Histone mutation, one of the hallmarks of DMGs, is the H3 K27M mutation that results in the loss of H3 K27 trimethylation. It is a mutant form of H3.3 or H3.1 that results from a specific genetic alteration in the H3F3A or HIST1H3B/C genes. The H3 K27M mutation leads to a global decrease in H3 K27me3, which is a histone modification that is normally associated with transcriptional repression. H3 K27M has been shown to inhibit the activity of the PRC2 complex, which is responsible for adding the H3 K27me3 modification to histones. This results in a loss of gene silencing, leading to an increased expression of genes that are normally repressed. This altered gene expression can contribute to the development and progression of DMGs [10]. On the other hand, H3 K27me3 is a histone modification that is normally associated with transcriptional repression. It is added to histones by the PRC2 complex, which includes the EZH2 protein. H3 K27me3 marks regions of the genome that are silenced, and its loss can lead to the activation of normally repressed genes. H3 K27me3 is also important for maintaining chromatin structure and regulating DNA replication and repair. A loss of H3 K27me3 due to H3 K27M mutation or other alterations can contribute to chromatin instability and genomic instability [12,14,30].

PDGFRA is a gene that encodes for a protein involved in cell growth and proliferation. Alterations in PDGFRA, such as amplifications or mutations, have been identified in a subset of DMGs and may contribute to tumor growth and progression [21,31,32].

CDKN2A and CDKN2B are two genes that encode proteins involved in regulating the cell cycle. Loss of function mutations or deletions of these genes are common in DMGs and are associated with a poorer prognosis [21,31].

ACVR1 is a gene that encodes for a protein involved in the regulation of bone growth and development. Mutations in ACVR1 have been identified in a subset of DMGs and may contribute to tumor development and progression [27,33].

MICA is a gene that encodes for a protein involved in the immune response. Alterations in MICA expression have been identified in DMGs and may contribute to immune evasion by tumor cells [34].

Alternative lengthening of telomeres (ALT) is a telomere maintenance mechanism that allows cancer cells to maintain their telomeres without the activation of telomerase, the enzyme responsible for telomere elongation in the majority of cancers. Instead, ALT utilizes a recombination-based mechanism to elongate telomeres by copying and recombining DNA sequences from other chromosomes. Studies have shown an association between ALT and certain types of tumors, including some gliomas. However, the exact prevalence of ALT in DMGs is still under investigation [35].

TP53 is a tumor suppressor gene that plays an important role in regulating the cell cycle, DNA repair, and apoptosis. Aberrant expression or loss of TP53 has been implicated in the development and progression of several types of cancer, including DMGs [36].

MAPK pathway is a signal transduction pathway that plays a critical role in the regulation of cell growth, proliferation, differentiation, and survival. Aberrant activation of the MAPK pathway has been implicated in the development and progression of several types of cancer, including DMGs. Mutations in genes that activate the MAPK pathway, such as BRAF, have been identified in a subset of DMGs [31,36,37,38].

Epidermal Growth Factor Receptor (EGFR) is a cell surface receptor protein involved in regulating cell growth and survival. It binds to ligands like EGF and TGF-alpha, leading to the activation of downstream signaling pathways that control cell proliferation, differentiation, and migration. Mutations in the EGFR gene, such as EGFRvIII, can result in a continuously active form of the receptor, promoting uncontrolled cell growth and has been reported to be associated with DMGs [39].

ATRX is a gene that encodes for a chromatin remodeling protein that plays a critical role in maintaining telomere length and heterochromatin integrity. The loss of ATRX function leads to alterations in telomere length and chromatin structure, which may contribute to tumor development and progression [40].

Phosphatidylinositol-4,5-bisphosphate 3-kinase catalytic subunit alpha (PIK3CA) is a gene that encodes a protein called PI3K-alpha, which is a key component of the PI3K/AKT/mTOR signaling pathway. This pathway plays a crucial role in regulating cell growth, proliferation, survival, and metabolism. Mutations in the PIK3CA gene have been implicated in various types of cancers, including gliomas. In the context of DMGs, studies have identified PIK3CA mutations in a subset of these tumors. It is important to note that PIK3CA mutations are not as frequently observed in DMGs compared to other molecular alterations such as H3 K27M mutations. The exact prevalence and significance of PIK3CA mutations in DMGs are still being investigated, and their specific role in the pathogenesis of these tumors is not yet fully understood [41].

Telomerase Reverse Transcriptase (TERT) is a gene that encodes the catalytic subunit of telomerase, an enzyme responsible for maintaining the length of telomeres—the protective caps at the ends of chromosomes. Mutations in the TERT gene can lead to abnormal activation of telomerase, allowing cancer cells to continually divide and evade the normal limits on cell replication. TERT mutations in DMGs are particularly relevant because they are associated with a poorer prognosis. Studies have shown that DMGs harboring TERT mutations tend to have a more aggressive clinical course, shorter overall survival, and higher resistance to therapy compared to those without TERT mutations [41].

Myeloid Cell Leukemia 1 (MCL1) is a gene that encodes a protein involved in the regulation of cell survival and apoptosis (programmed cell death). It belongs to the Bcl-2 family of proteins, which play a critical role in maintaining the balance between cell survival and cell death. Studies have shown that MCL1 is frequently overexpressed in DMGs, meaning that there is an increased production of the MCL1 protein compared to normal cells. This overexpression of MCL1 has been associated with increased cell survival and resistance to cell death mechanisms, contributing to the aggressive nature of DMGs. The dysregulation of MCL1 in DMGs suggests that it may be a potential therapeutic target. Strategies aimed at inhibiting MCL1 such as small molecule inhibitors or RNA interference-based approaches have shown promise in preclinical studies as a means to enhance tumor cell death and sensitize DMGs to other treatment modalities, such as radiation or chemotherapy [42].

Isocitrate Dehydrogenase (IDH) mutations are genetic alterations commonly observed in various types of gliomas, including diffuse midline gliomas (DMGs). IDH mutations occur in the IDH1 or IDH2 genes, which encode enzymes involved in cellular metabolism. DMGs with IDH mutations tend to occur in older patients compared to those without. These mutated DMGs may also exhibit different histological features and gene expression profiles. Additionally, the presence of IDH mutations in DMGs has been suggested to confer a more favorable prognosis. Compared to DMGs without IDH mutations, patients with IDH-mutant DMGs may have improved overall survival and respond differently to certain treatment modalities. However, it is important to note that the overall prognosis for DMGs remains poor, regardless of IDH mutation status [43].

## 4. Clinical Presentation and Prevalence

The prevalence of DMGs varies depending on the specific midline structure affected and the age group. In children, DMGs account for 10–20% of all pediatric brain tumors. DMGs in other locations are rare and the approximate prevalence is difficult to provide [44].

Presenting neurological symptoms include persistent headaches, which varies in severity and worsens over time, dizziness, ataxia with difficulty in coordination, such as stumbling or difficulty walking in a straight line, weakness or paralysis on one side of the body, difficulty speaking, such as slurred speech or difficulty finding the right words, changes in vision or hearing, such as blurred vision or hearing loss, and seizures, which may be focal or generalized [16,45].

In addition, behavioral changes can also be observed in individuals with DMGs. These may include emotional or personality changes such as alterations in mood, behavior regulation, mood swings, irritability, and cognitive decline, including problems with memory, attention, and problem solving. These behavioral changes can be attributed to the tumor’s location in critical areas of the brain that regulate behavior, emotions, and cognitive function, as well as the infiltrative nature of DMGs that disrupts normal brain tissue function [46].

It is important to note that the clinical features of DMGs can vary depending on the location and grade of the tumor, and the specific clinical manifestations may be related to the area affected by the DMG [47]. For example, DMGs located in the brainstem often present with distinct symptoms such as difficulty swallowing, facial weakness, and problems with eye movements, indicating cranial nerve involvement [48].

Although metastasis is not a typical feature of DMGs, leptomeningeal dissemination or periventricular spread are reported [49]. A study by Tauziède-Espariat et al. reported a case of a DMG patient with a histone H3 K27M mutation who developed leptomeningeal dissemination [50]. Moreover, a study by Al Sharie et al. reported osseous metastasis to the vertebrae and the right iliac bone [51]. These metastatic features of DMGs can make the management of DMGs more challenging and impact treatment decisions. Therefore, it is important for clinicians to be aware of these features and consider them in the diagnostic and therapeutic management of DMGs [44].

## 5. Radiological Features

### 5.1. Magnetic Resonance Imaging (MRI):

MRI is the imaging modality of choice for evaluating DMGs due to its excellent soft tissue contrast and multiplanar imaging capabilities [52]. DMGs typically appear as infiltrative and diffusely expanding lesions on MRI, often involving the midline structures of the brain. The findings may vary depending on the location and histological subtype of DMG [53]. On T1-weighted imaging (T1WI), DMGs typically appear hypointense due to their dense cellularity and lack of necrosis. However, in some cases, DMGs may show hyperintensity, particularly in the presence of hemorrhage or cystic changes. On T2-weighted imaging (T2WI), DMGs typically appear hyperintense due to their infiltrative nature and increased water content. The tumor may show a diffuse, ill-defined margin with indistinct borders, making it challenging to delineate from the surrounding normal brain tissue. Moreover, fluid-attenuated inversion recovery (FLAIR) images are particularly useful in identifying the extent of tumor infiltration as DMGs typically show hyperintensity on FLAIR imaging, allowing for better visualization of the tumor margins. Additionally, diffusion weighted imaging (DWI) can be helpful in evaluating the cellular density of the tumor, as DMGs typically show restricted diffusion due to their high cellularity. Restricted diffusion on DWI can be helpful in distinguishing DMGs from other brainstem lesions, such as demyelinating lesions or inflammatory processes. Finally, DMGs may or may not show contrast enhancement on MRI. In some cases, DMGs may demonstrate minimal to no enhancement, while in other cases, they may show variable enhancement patterns, such as patchy or ring-like enhancement [54,55,56,57].

**Figure 2:**
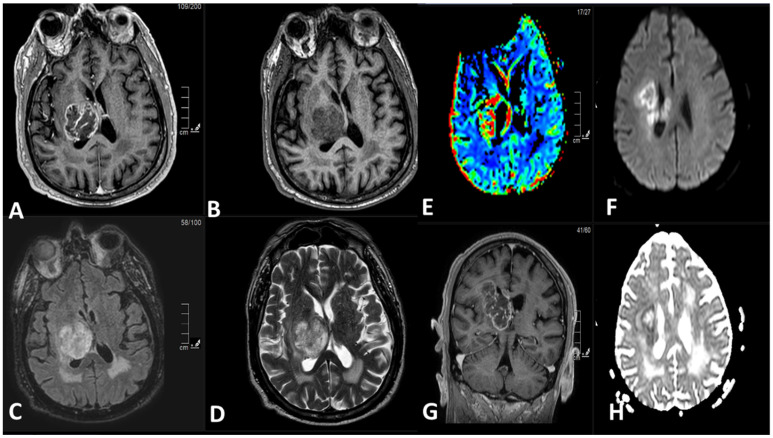
(**A**) Axial T1-weighted post IV contrast sequence. (**B**) Axial T1-weighted pre-IV contrast sequence. (**C**) Axial FLAIR sequence. (**D**) Axial T2-weighted sequence. (**E**) Dynamic susceptibility contrast (DSC) MR perfusion images, cerebral blood volume (CBV). (**F**) Diffusion weighted images (DWI), b 1000. (**G**) Coronal T1-weighted post IV contrast sequence. (**H**) Apparent diffusion coefficient (ADC) map.

Figure 2 demonstrates an example of an intra-axial mass lesion in the right thalamus. It shows an irregular border, hypointense signal on T1-weighted sequence, heterogeneous signal on T2-weighted sequence with intermediate and hyperintense signal areas, and hyperintense FLAIR signal. The lesion shows peripheral irregular contrast enhancement with a rim of diffusion restriction and hyperperfusion. It results in a mass effect upon the body and trigone of the right lateral ventricle. There is very mild perilesional edema. The confluent T2, FLAIR hyperintense white matter signal in periventricular regions correlates most with sequelae of microvascular ischemic change. The coronal images show the craniocaudal extent of the lesion; there is a mass effect upon the ventricular system with a small tumor component within the corpus callosum.

**Figure 3:**
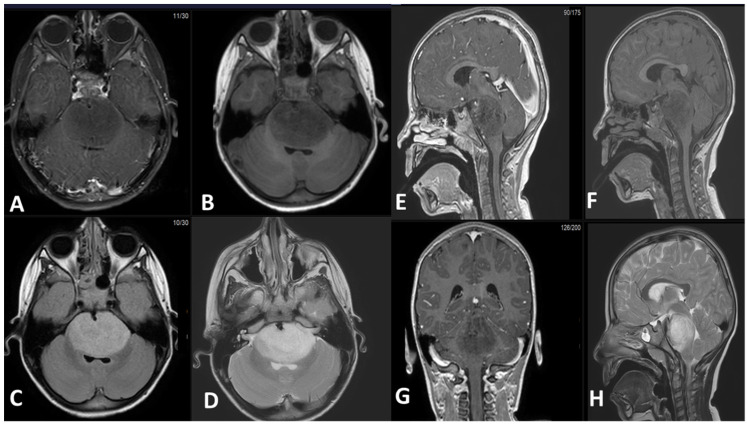
(**A**) Axial T1-weighted post IV contrast fat saturated sequence. (**B**) Axial T1-weighted pre-IV contrast sequence. (**C**) Axial FLAIR sequence post IV contrast. (**D**) Axial T2 TSE sequence. (**E**) Sagittal T1-weighted post IV contrast sequence. (**F**) Sagittal T1-weighted pre-IV contrast sequence. (**G**) Coronal T1-weighted post IV contrast sequence. (**H**) Sagittal T2 TSE sequence. FLAIR (fluid attenuation inversion recovery). TSE (turbo spin echo).

Figure 3 demonstrates an example of DMG arising from the pons. There is an expansible mass involving the brainstem, centered in the pons, obliterating the prepontine cistern and encasing the basilar artery. The mass shows hypointense signal on T1-weighted sequence, hyperintense signal on T2 and FLAIR sequences, and is non-contrast enhancing. It compresses the fourth ventricle. There is no proximal ventricular dilatation in this patient. The sagittal and coronal images show the proximal and distal extension of the mass to the pontomesencephalic and pontomedullary junctions, showing a somewhat indistinct border.

**Figure 4:**
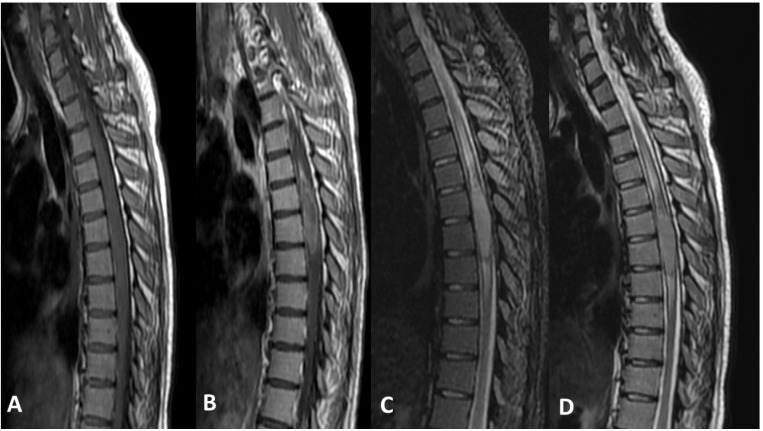
(**A**) Sagittal T1-weighted pre-IV contrast sequence of the dorsal spine. (**B**) Sagittal T1-weighted post IV contrast sequence of the dorsal spine. (**C**) Sagittal STIR sequence of the dorsal spine. (**D**) SagittalT2-weighted sequence of the dorsal spine.

Figure 4 demonstrates an example of DMG arising from the spinal cord. There is an intramedullary mass lesion in the mid-dorsal spinal cord. The mass is expansile, showing almost isointense signal on T1 and T2-weighted sequences and mild STIR hyperintense signal with heterogeneous post contrast enhancement. The mass extends for almost four vertebral levels craniocaudally, with moderate cord edema proximal and distal to the lesion.

It is worth noting that DMG in general, and DIPG in particular, is most commonly diagnosed solely based on imaging criteria, with biopsy often reserved for tumors with atypical imaging features [58]. Along with the classical clinical presentation, the typical MRI appearance allows to establish the diagnosis without resorting to a biopsy diagnosis [59,60]. However, a definitive diagnosis of DMGs might require histopathological confirmation if the radiological appearances deviate from the usual patterns. Thus, imaging findings should be interpreted in conjunction with clinical presentation and other diagnostic tests to arrive at an accurate diagnosis [53].

### 5.2. Positron Emission Tomography (PET)

PET imaging using radiotracers, such as 18F-fluorodeoxyglucose (FDG) or 11C-methionine (MET), can provide additional information about the metabolic activity and cellular proliferation of DMGs [61]. DMGs typically show increased FDG uptake due to their high metabolic activity and cellular proliferation. FDG-PET can be useful in assessing the extent of tumor infiltration, evaluating treatment response, and distinguishing DMGs from other brainstem lesions, such as inflammatory processes or radiation necrosis [62]. On the other hand, MET-PET can be particularly helpful in distinguishing DMGs from other brainstem lesions, as DMGs typically show increased MET uptake due to their high cellular proliferation rate. MET-PET can also provide valuable information for biopsy targeting and treatment planning [63].

DOPA-PET, which stands for Dihydroxyphenylalanine Positron Emission Tomography, is a specialized imaging technique that utilizes a radiolabeled form of the amino acid DOPA to detect and visualize brain tumors, including gliomas [64]. DOPA-PET is particularly useful in the context of DMGs, as these tumors often exhibit high levels of amino acid transporters, including those responsible for transporting DOPA [65]. This imaging technique can provide valuable information about the location, extent, and metabolic activity of the tumor. It is especially beneficial for cases where standard imaging methods like MRI may not provide a clear view of the tumor or its borders [65].The radiotracer used in DOPA-PET emits positrons, which are detected by the PET scanner. By analyzing the distribution of positron emissions, this information can aid in treatment planning, assessing treatment response, and monitoring disease progression [66,67].

## 6. Histological Features

Histologically, DMGs are characterized by their diffuse growth pattern and the presence of malignant astrocytic or oligodendroglial-like cells [68]. These cells have distinct histologic features, including increased cellularity, nuclear atypia, and mitotic activity [69]. In addition, DMGs often exhibit a perivascular pseudorosettes pattern, where tumor cells surround blood vessels [69]. The grading of DMGs has evolved over time. Whereas prior to 2016 classification, tumors were traditionally classified as grade-2 diffuse astrocytoma, grade-3 anaplastic astrocytoma, and glioblastoma, depending on the absence or presence of a constellation of microscopic features including mitosis, microvascular proliferation and/or necrosis. More recently, the diagnosis of DMG would imply a grade 4 tumor, regardless of the aforementioned microscopic features, if mutations can be identified [70]. Traditional grading can still be used in DMGs that do not exhibit diagnostic molecular features. Interestingly, some correlation between the morphology and molecular signature of the tumor has been described. For example, the H3 K27M was detected exclusively in WHO grade 2–4 tumors with astrocytoma morphology, whereas H3.1 K27M-, *ACVR1*, and ALT mutations were detected only in WHO grade 3–4 astrocytoma [36]. Cases with embryonal morphology (formally known as primitive neuro-ectodermal tumors; ex-PNET) and histology have been reported and show *TP53* mutation but were negative for GFAP immunostain without H3 K27 mutation [36].

Several molecular and cytogenetic alterations have been identified in DMGs that can aid in their diagnosis and classification. These include mutations in the H3F3A or HIST1H3B/C genes, which result in the H3 K27M mutation, and thus, immunoreactivity with the H3 K27M immunostain and the loss of H3 K27me3 staining on immunohistochemistry [10,14]. In addition, DMGs often exhibit mutations or alterations in the *TP53* and *ATRX* genes [71].

Other molecular alterations that have been identified in DMGs include mutations or amplifications of the EGFR, *PDGFRA*, and *ACVR1* genes, as well as overexpression of EZHIP and Protein Phosphatase, Mg^2+^/Mn^2+^ 1ependent 1D (PPM1D). These alterations can have implications for the treatment and clinical management of patients with DMGs [72,73,74,75,76].

Furthermore, the presence of *CDKN2A*/B homozygous deletion in DMGs has been associated with distinct molecular subtypes of these tumors, which are characterized by alterations in several signaling pathways, including the phosphoinositide 3-kinase (PI3K)/mTOR pathway. These subtypes are associated with distinct clinical presentations and prognoses compared to other subtypes of DMGs [76,77,78].

**Figure 5:**
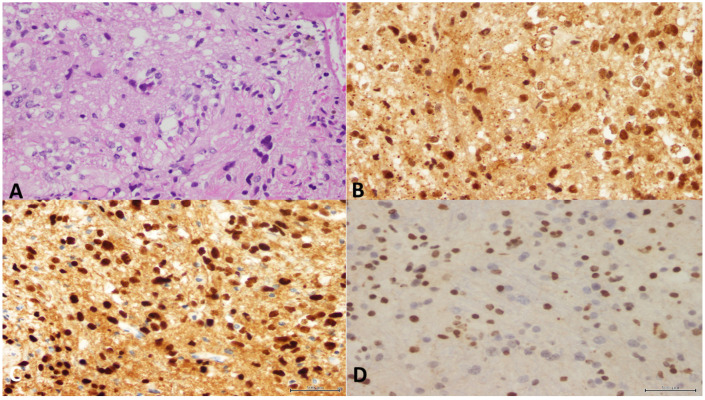
A 52-year-old woman presented with disorientation, right ear pain, and mood changes of 2 months duration. The overall appearance for the radiologist was suggestive of low-grade glioma with a possibility of high-grade cystic component. Accordingly, a stereotactic biopsy of the left thalamic lesion was performed. (**A**) A moderately cellular tumor with pleomorphic cells and scattered mitotic figures (H&E, X20). (**B**) *ATRX* immunostain showed retained nuclear stain. (**C**) H3 K27M showed positive nuclear staining. (**D**) H3 K27me3 showed loss of nuclear stain of the infiltrating tumor cells. The final diagnosis was DMG, H3 K27-altered, CNS WHO grade-4.

**Figure 6:**
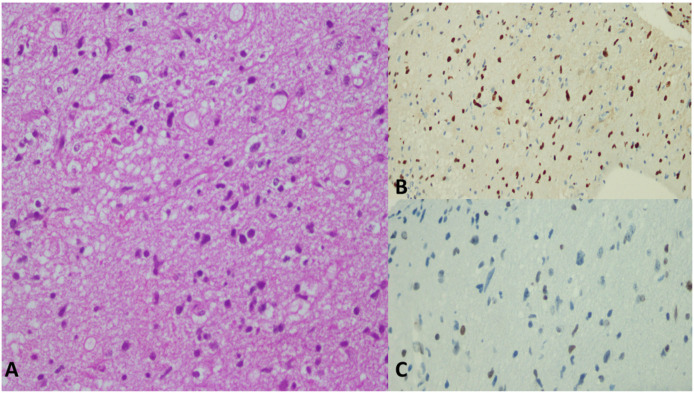
A 7-year-old female patient with a midbrain mass invading the pons and the thalamus. (**A**) High power from the abnormal area with mild increase in cellularity as well as scattered atypical cells. No mitotic figures, microvascular proliferation or necrosis could be appreciated. The case was diagnosed in 2014 as grade-2 fibrillary astrocytoma. Recently, further immunostains were performed. (**B**) The atypical tumor cells are positive for H3 K27M immunostain (normal cells serve as the negative internal control). (**C**) Immunostain for H3 K27me3 showed loss of nuclear stain in the infiltrating atypical tumor cells (normal cells serve as the internal positive control). The new findings supported reclassification of the tumor as DMG, H3 K27-altered, CNS WHO grade 4.

**Figure 7:**
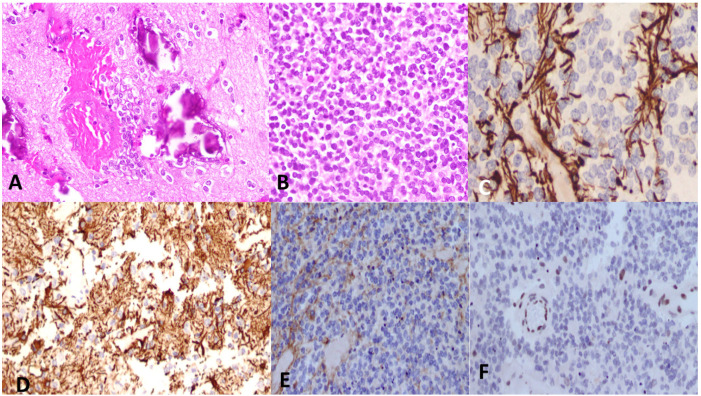
A spinal cord tumor in a 14-year-old male patient. (**A**) On H&E, there was aggregation of small cells in a fibrillary background, with calcifications. (**B**) In other areas, the tumor was composed of compact proliferation of small cell. (**C**) GFAP immunostain was negative in the tumor cells. (**D**) Synaptophysin was positive. (**E**) The H3 K27M immunostain showed no nuclear stain, while (**F**), H3 K27me3, showed loss of nuclear stain (endothelium serves as positive internal control). The final diagnosis was DMG, H3 K27-altered, CNS WHO grade-4.

Figure 5, Figure 6 and Figure 7 demonstrate histopathological features of DMGs in different patients.

## 7. The Role of Biopsy in DMGs

While the diagnosis of DMG is usually based on clinical and radiological features, such as midline location and diffuse infiltrative growth pattern, biopsy can play a critical role in confirming the diagnosis of DMGs and providing molecular information that can guide treatment decisions [58]. Specifically, biopsies can rule out other pathologies that are treated differently, like embryonal tumors (i.e., ex-PNET) [36]. Moreover, biopsies are needed to confirm the presence of the H3 K27M mutation, which is a defining cornerstone for accurate diagnosis and classification of DMGs [79]. In addition, biopsy can provide other molecular information that can help determine the appropriate treatment strategy. For example, the co-occurrence of other genetic alterations, such as *TP53* mutations or amplification of the *PDGFRA* gene, may influence outcomes in DMG and guide the selection of clinical trials or targeted therapies that specifically target these mutations [33,80,81].

It is worth noting that several groups have explored different biopsy techniques and their applications in DMGs. These techniques may include stereotactic biopsy, which uses image-guided technology to target the tumor site precisely, minimizing damage to healthy brain tissue [82]. This technique is known for its accurate diagnosis as it provides a minimally invasive method to obtain tissue samples from these hard-to-reach areas [83]. By precisely targeting the tumor using imaging guidance, such as MRI or CT, a small sample of the tumor can be extracted for pathological analysis allowing pathologists to determine the specific tumor type and grade [84], as well as molecular characterization of the tumor [85,86]

Liquid biopsy, a relatively new technique, involves the analysis of tumor-derived biomarkers in body fluids such as blood, cerebrospinal fluid (CSF), or urine [87]. This approach has emerged as a promising tool for the diagnosis and monitoring of cancer, including brain tumors such as DMGs [88]. Liquid biopsy can detect cell-free DNA (cfDNA), circulating tumor DNA (ctDNA), and other tumor-derived products in the bloodstream or CSF [89]. These biomarkers can be used to monitor the response to treatment, detect disease recurrence, and potentially guide treatment decisions [90,91]. One of the advantages of liquid biopsy is that it is less invasive than traditional biopsy procedures, which may be associated with risks such as bleeding or infection [92]. Liquid biopsy can be performed using a simple blood draw, which is generally well tolerated by patients. In addition, liquid biopsy can be repeated more frequently than traditional biopsy, allowing for more frequent monitoring of disease status [93,94]. Several studies have investigated the use of liquid biopsy in the diagnosis and monitoring of brain tumors, including DMG [95]. For example, one study found that ctDNA could be detected in the plasma of patients with DMG and that ctDNA levels were associated with disease burden and survival. Furthermore, it was found that analysis of cfDNA in the CSF could provide useful diagnostic information in patients with DMG, particularly in cases where the diagnosis is uncertain or biopsy is not feasible [96].

While liquid biopsy holds promise as a tool for the diagnosis and monitoring of DMG, there are some limitations to its use including that the sensitivity of liquid biopsy can vary depending on the tumor type, location, and molecular features [97]. In addition, the identification of actionable targets from liquid biopsy requires careful interpretation and validation, as ctDNA can be derived from non-malignant cells or non-tumor tissues [98].

## 8. Treatment Options

### 8.1. Chemotherapy

Several chemotherapy agents have been used for DMGs, including temozolomide, carboplatin, and etoposide [99,100,101].

Temozolomide is an alkylating agent that works by damaging DNA and preventing tumor cells from dividing. It is the most commonly used chemotherapy drug for DMGs and has been shown to be moderately effective in clinical trials. However, its efficacy is limited, and responses are often short-lived, with most patients experiencing disease progression within a few months [99,102].

Carboplatin is a platinum-based chemotherapy drug that also works by damaging DNA and preventing tumor cell division. It has been used in combination with other chemotherapy drugs for the treatment of DMGs, but its efficacy is limited and often associated with significant side effects [103,104,105].

Etoposide is a topoisomerase inhibitor that works by preventing the replication of DNA. It has been used in combination with other chemotherapy agents for the treatment of DMGs, but its efficacy is limited, and responses are often short-lived [106,107].

Despite the use of chemotherapy for DMGs, treatment outcomes for these tumors remain poor. The effectiveness of chemotherapy is often limited by the intrinsic resistance of DMGs to these agents, as well as by the blood-brain barrier, which can prevent chemotherapy drugs from reaching the tumor cells in sufficient quantities [107,108,109].

Furthermore, chemotherapy for DMGs is often associated with significant side effects, including fatigue, nausea, vomiting, hair loss, and an increased risk of infections [110]. In addition, these tumors often recur rapidly after treatment, and the use of chemotherapy as a salvage therapy is often limited by cumulative toxicity and poor tolerance [111].

### 8.2. Radiotherapy

Radiotherapy is a mainstay of treatment for DMGs, especially for those that cannot be completely surgically resected [78]. Radiation therapy utilizes high-energy X-rays or other forms of radiation to target and destroy cancer cells in the brain [112]. In DMGs, radiotherapy is typically delivered to the entire brain and spinal cord, a technique known as craniospinal irradiation, to target any potential areas of tumor spread [113].

Radiotherapy can be administered using different techniques, including conventional external beam radiation therapy, intensity-modulated radiation therapy (IMRT), and proton beam therapy. IMRT and proton beam therapy are more advanced techniques that can more precisely target the tumor while sparing normal brain tissue [78].

The optimal timing for radiotherapy in DMGs is a topic of ongoing research. Historically, radiotherapy was delayed until disease progression or until the child was at least 3 years old due to concerns about the potential adverse effects of radiation on the developing brain [114]. However, more recent studies have shown that early initiation of radiotherapy may lead to better outcomes, including longer survival [115,116,117].

The standard dose of radiation for DMGs is typically around 54–60 Gy and may reach up to 66–70 Gy based on case characteristics; it is delivered over the course of several weeks [118,119,120,121].

Despite the effectiveness of radiotherapy in treating DMGs, there are potential short- and long-term side effects that need to be carefully considered. Short-term side effects can include fatigue, headaches, nausea, and hair loss. Long-term side effects can include cognitive impairment, endocrine dysfunction, and radiation-induced secondary tumors. Therefore, close follow-up with a multidisciplinary team, including neuro-oncologists and radiation oncologists, is critical to monitor and manage any potential side effects [122,123].

Re-irradiation can be considered in some cases of DMGs when the tumor recurs or progresses after initial treatment [124]. However, the decision to undergo re-irradiation depends on several factors, including the patient’s overall health, location and size of the tumor, previous radiation dose, interval between initial radiation and recurrence, and potential risks and benefits of further treatment [44].

Since DMGs have a high tendency for recurrence within the area of treatment, re-irradiation becomes an option for palliation [125]. It is generally advised to wait at least 3–6 months between radiation treatments [125]. The radiation dose in the re-irradiation scenario is in the range of 24–25 Gy administered over 10–12 fractions over 2–2.5 weeks [126,127]. When compared to no radiation at the time of recurrence, treatment has been shown to improve median survival. It is also still well tolerated, with the majority of patients experiencing further clinical relief [128].

### 8.3. Targeted Therapy and Immunotherapy

One of the key molecular targets in diffuse midline gliomas is the histone protein. The H3F3A and HIST1H3B alterations provide opportunities for targeted therapies aimed at correcting the aberrant epigenetic modifications and restoring normal gene expression patterns [129]. For example, inhibitors of specific enzymes involved in the methylation of histone proteins, such as EZH2 and DOT1L, have shown promise in preclinical studies and early-phase clinical trials [130,131].

Another important molecular target in diffuse midline gliomas is *PDGFRA*. *PDGFRA* is frequently overexpressed in DMGs and is associated with tumor growth and invasion. Inhibitors of *PDGFRA*, such as imatinib, dasatinib, and crenolanib, have shown promising results in preclinical studies and early-phase clinical trials, with evidence of tumor regression and improved survival in some cases [132,133]. Additionally, other signaling pathways such as the EGFR [134,135] and the PI3K/Akt/mTOR pathway have also been implicated in the pathogenesis of DMGs and are being explored as potential targets for therapy [136,137].

The exploration of the role of EGFR signaling in childhood DIPG led to the use of nimotuzumab, an anti-EGFR monoclonal antibody [138]. A pilot phase 2 study was conducted, combining nimotuzumab with concurrent radiotherapy and vinorelbine, along with re-irradiation at relapse, with positive responses seen in the majority. The nimotuzumab/vinorelbine combination was well tolerated with minimal side effects. Survival rates at different intervals were recorded, demonstrating promising outcomes [139].

Immunotherapy has also gained attention as a potential targeted therapy for DMGs. Immune checkpoint inhibitors, such as nivolumab, pembrolizumab, durvalumab, avelumab, and ipilimumab, which target programmed cell death protein 1 (PD-1) and its ligand (PD-L1), have shown encouraging results in other types of gliomas and have been explored in DMGs as well [17,140]. However, the effectiveness of immunotherapy in DMGs is still being investigated, as these tumors are known to have an immunosuppressive microenvironment that may limit the response to immune checkpoint inhibitors [141,142]. Combining immunotherapy with other targeted therapies or radiation therapies may offer potential synergistic effects and is an area of ongoing research [143].

In addition to molecular targets, genetic alterations in DMGs, such as mutations in genes like *TP53*, *ACVR1*, and *ATRX*, have also been identified as potential therapeutic targets. For example, studies have shown that restoration of wild-type *TP53* function using small molecule drugs or gene editing approaches can lead to tumor regression in DMGs [144]. Similarly, inhibitors of *ACVR1*, a receptor kinase that is frequently mutated in DMGs, have shown promise in preclinical studies and early-phase clinical trials [33,75]. Furthermore, *ATRX* mutations have been associated with increased sensitivity to certain chemotherapeutic agents, such as temozolomide, suggesting potential therapeutic strategies based on the genetic profiling of tumors [145].

Several ongoing clinical trials are currently evaluating the safety and efficacy of targeted therapies in diffuse midline gliomas. For example, clinical trials investigating inhibitors of histone methylation enzymes, such as tazemetostat (NCT03213665) and GSKJ4 (NCT03858999), are underway. Other trials are evaluating inhibitors of *PDGFRA*, such as imatinib (NCT03433104) and crenolanib (NCT02847429), in DMGs. Additionally, clinical trials exploring combinations of targeted therapies, immunotherapies, and radiation therapy are also ongoing, aiming to exploit potential synergistic effects and improve treatment outcomes for these aggressive tumors (NCT03696355, NCT03566199).

ONC201, also known as TIC10, is a small molecule that can be taken orally and has the ability to enhance the activity of TNF-related apoptosis-inducing ligand (TRAIL). By doing so, it restores the ability of cancer cells to undergo programmed cell death and inhibits tumor growth. This compound can also cross the blood-brain barrier, making it a potential treatment option for tumors in the brain, which is a limitation of traditional chemotherapy. ONC206, a similar compound to ONC201, is a more potent version that induces cancer cell death [146,147].

A phase 2 clinical trial conducted by Arrillaga-Romany et al. examined the effectiveness and tolerability of ONC201 as a monotherapy. The treatment was found to be well tolerated, with no significant adverse effects leading to treatment discontinuation. Tumor response was evaluated using MRI scans, and the overall response rate was 30%, with an average duration of response being approximately 52.7 weeks. Another patient with stable disease for over 12 months showed partial response (NCT02525692). Another ongoing trial by Arrillaga-Romany et al. is investigating the safety and efficacy of ONC206 in a Phase 1 study with patients diagnosed with a specific type of brain tumor. Results of this trial are pending publication (NCT04541082).

Arrillaga-Romany et al. are also conducting a Phase 3 clinical trial called ACTION, which aims to evaluate the effectiveness and safety of ONC201 in individuals with a specific genetic mutation in their brain tumors. This trial follows a randomized, double-blind, and placebo-controlled design, and its primary objectives include evaluating overall survival and progression-free survival, along with assessing safety, efficacy measures, quality of life, and biomarkers (NCT05580562).

Gardner et al. conducted a clinical trial with pediatric patients to assess the safety of administering ONC201 at the recommended dose for adults, adjusted for body weight. The trial included patients who had previously received radiation therapy. The treatment was well tolerated, with no dose-limiting toxicity. The pharmacokinetic profiles of ONC201 in pediatric patients were similar to those observed in adults. Among a subgroup of patients who received ONC201 after radiation, 45% continued therapy, showing promising survival outcomes (NCT03416530).

In a recent study by Venneti et al. published in Cancer Discovery, ONC201 showed significant antitumor activity in both non-recurrent and recurrent tumors, with median overall survival of 21.7 months for patients with non-recurrent tumors and 9.3 months for patients with recurrent tumors. The objective response rate was 36.8% for patients with non-recurrent tumors and 40.9% for patients with recurrent tumors. Reduced baseline normalized relative cerebral blood volume (nrCBV) was associated with improved OS and PFS, suggesting that nrCBV may be a potential biomarker of improved response to ONC201. Overall, the results of this trial show that ONC201 is a safe and effective treatment for H3K27M-DMG, and further clinical trials are needed to confirm these findings and to identify the best way to use ONC201 [148].

ONC201 and ONC206 bind to ClpP, a protease that is involved in the degradation of misfolded proteins in the mitochondria, in a unique manner that is distinct from other ClpP activators [149]. This binding results in a conformational change in ClpP that increases its proteolytic activity. ONC201 and ONC206 have also been shown to promote the oligomerization of ClpP, which further enhances its activity [149]. This increase in ClpP activity is associated with a decrease in the levels of misfolded proteins in the mitochondria [149].

Moreover, it has been shown that ONC201 and ONC206 affect mitochondrial activity in cancer cells by upregulating the expression of genes involved in mitochondrial biogenesis and function [150]. This increase in mitochondrial activity leads to increased cellular energy production and metabolism [150].

Furthermore, ONC201 and ONC206 have been shown to activate the Integrated Stress Response (ISR) in a number of different cell types [151]. This activation is thought to be due to the increased mitochondrial Reactive Oxygen Species (ROS) production that is caused by these drugs [151]. The activation of the ISR may contribute to the antitumor activity of ONC201 and ONC206, thus leading to cell cycle arrest and apoptosis in tumor cells [151].

A thorough search using the portal ClinicalTrials.gov was conducted to include all clinical trials examining DMG and DIPG that are currently available (Table S1). A total of 131 clinical trials were identified, involving 30,445 participants, with the aim of addressing the unique challenges associated with DMG and DIPG tumors. The trials were categorized into different phases to assess the progression of research and development. Of the identified trials, 62 were in phase 1, 22 trials were in phase 2, 24 trials were classified as phase 1/phase 2 trials, and a smaller number of trials, specifically 6, were in phase 3. The study types of the identified trials varied, reflecting different approaches to understanding and managing DMG tumors. Out of the total trials, 120 were classified as interventional studies, 9 trials were categorized as observational studies, and 2 trials were designated as expanded access studies with an intermediate-sized population. Among the countries involved in conducting the identified clinical trials, the United States was most prominent, with 101 trials taking place in the country.

It is important to note that targeted therapies for DMGs are still in the early stages of development, and their long-term safety and efficacy are yet to be determined. Challenges such as blood-brain barrier penetration, tumor heterogeneity, and resistance mechanisms may affect the success of targeted therapies in this context [22,152,153,154]. However, the identification of specific molecular targets and genetic alterations has opened up new possibilities for personalized and precision medicine approaches, and ongoing research and clinical trials hold promise for improved treatment options in the future.

An interesting clinical trial by a Stanford team investigated the use of GD2-CAR T cells immunotherapy for DMGs [155]. The study showed promise but also presented risks due to potential brain inflammation. The toxicity associated with GD2-CAR T cell infusions was manageable with supportive care. Patients experienced inflammation-related symptoms in the CNS, specifically, Tumor Inflammation-Associated Neurotoxicity (TIAN), which could be serious and requires prompt management. TIAN fell into two categories: one involving intracranial pressure and space issues and the other involving temporary dysfunction of affected CNS structures. Despite neurotoxic symptoms, patients did not show signs of harmful effects on normal neural tissues expressing GD2. The study supported the safety and effectiveness of targeting GD2 with CAR T cells. Three out of four patients showed benefit from GD2-CAR T cell treatment, with improvement in neurological function. Intracranial administration of the therapy was associated with less systemic toxicity and showed potential for enhancing the immune response within the CNS. The positive early results pave the way for further optimization of GD2-CAR T cell therapy for these deadly CNS cancers tumors.

## 9. Key Determinants of Drug Resistance

These tumors are often associated with poor prognosis and limited treatment options, partly due to their intrinsic resistance to therapies [23]. The key determinants of drug resistance in DMGs are plentiful, including heterogeneity, the blood-brain barrier (BBB), and genetic and epigenetic alterations, among others.

DMGs are highly heterogeneous tumors, both in terms of genetic mutations and cellular composition. This heterogeneity can lead to subpopulations of tumor cells that respond differently to treatment, making it challenging to target all the resistant cells effectively [111]. The blood-brain barrier plays a critical role in the context of DMGs. The BBB restricts the entry of substances, including potentially therapeutic drugs, from the bloodstream into the brain, thereby posing a significant challenge for DMG treatment. The barrier’s selective permeability prevents many systemic therapies from reaching tumor cells effectively, leading to suboptimal drug concentrations within the tumor microenvironment. This limited drug delivery contributes to the intrinsic resistance of DMGs to treatment and underscores the need for innovative therapeutic strategies that can bypass or overcome the BBB to enhance the efficacy of interventions against these challenging tumors [156].

Genetic mutations: mutations in genes such as H3F3A, HIST1H3B, and *TP53* are commonly found in DMGs and can contribute to resistance by altering cellular processes that affect drug sensitivity, DNA repair, and apoptosis [157]. Epigenetic changes, on the other hand, including alterations in DNA methylation and histone modifications, can impact gene expression and cellular responses to treatment. These changes may lead to the activation of resistance pathways or the silencing of pro-apoptotic genes [158].

Other potential causes for the development of drug resistance include the dysregulation of signaling pathways, such as the PI3K/AKT/mTOR and MAPK/ERK pathways, which can promote cell survival and resistance to therapy. Aberrant activation of these pathways can render tumor cells less responsive to cytotoxic effects induced by treatment [159]. Some tumor cells within DMGs may possess stem cell-like properties, allowing them to self-renew and differentiate into various cell types. These cells can contribute to tumor regrowth after treatment and are often more resistant to therapies due to their inherent plasticity [160]. Tumor microenvironment, including interactions with immune cells, stromal cells, and extracellular matrix components, can influence drug resistance. Immunosuppressive factors in the microenvironment can hinder immune-mediated tumor clearance [161].

One interesting mechanism that might account for the resistance is the drug efflux pumps, whereby tumor cells develop mechanisms to pump out drugs from within the cell, reducing their intracellular concentrations and thereby decreasing their effectiveness. This drug efflux is often mediated by transporters like P-glycoprotein [162].

Finally DMG cells can activate DNA repair pathways, such as homologous recombination and non-homologous end joining, which enable them to repair DNA damage induced by chemotherapy or radiation, leading to treatment resistance [163].

## 10. Prognostic Factors

Several prognostic factors have been identified in the literature that can impact the outcome of patients with DMGs. One of the most significant prognostic factors is the histone H3 mutation status. Studies have shown that patients with DMGs harboring the K27M mutation in the histone H3.3 gene (H3F3A) have a worse prognosis compared to those without this mutation [164]. The presence of other genetic alterations, such as *TP53* mutations and amplification of the *PDGFRA* gene, has also been associated with poorer prognosis [73].

The age of the patient at diagnosis has also been identified as a prognostic factor. Younger age at diagnosis has been associated with better overall survival in several studies, with some reports suggesting that patients diagnosed at a younger age (e.g., less than 5 years) have a slightly better prognosis compared to older patients. However, the impact of age on prognosis may vary depending on the specific type of DMG and the associated genetic alterations [165,166,167].

Other reported factors include the extent of tumor resection and the response to treatment. Studies have shown that patients who undergo more extensive tumor resection tend to have better outcomes compared to those with residual tumor after surgery, which is often challenging and unamenable [144]. The response to treatment, including radiation therapy and chemotherapy, can also impact prognosis, as patients who respond well to initial treatment may have improved survival [44,111,168,169].

It is also suggested that patients with symptoms persisting for more than 3 months have a better prognosis than those with symptoms less than 3 months [2,170,171]. The absence of cranial nerve palsies at diagnosis and rapid improvement of neurological symptoms are also associated with better outcomes [169,172]. On the other hand, the presence of ring enhancement on MRI, restricted diffusion areas, and the presence of metastatic disease are all factors associated with poor prognosis for patients with DMGs [32,169,173].

It is worth noting that prognosis for DMGs remains poor overall, with median survival typically ranging from months to a few years, even with aggressive treatment approaches [29].

## 11. Conclusions

DMGs are an aggressive type of brain tumors that continue to pose significant treatment challenges for clinicians and researchers. However, ongoing research efforts are uncovering promising new insights into the biology of these tumors, which has resulted in major classification modification and is allowing the identification of new potential targets for therapy.

Precision medicine approaches, such as genomic sequencing and targeted therapies, might hold great potential for improving outcomes for patients with this fatal disease. Immunotherapies, including cell therapies, are also emerging as a potential treatment avenue, with research focusing on harnessing the immune system’s ability to fight cancer.

Interdisciplinary collaboration and data sharing are essential components in accelerating progress in the field of DMG research. By bringing together experts from diverse fields, researchers can combine their knowledge and resources to address complex challenges and develop innovative solutions.

## Data Availability

Not applicable.

## Supplementary Materials

The following supporting information can be downloaded at: https://www.mdpi.com/article/10.3390/cancers15194869/s1, Table S1: All currently available clinical trials examining DMG and DIPG.
