# Supplementary material for: Decoding Diffuse Midline Gliomas: A Comprehensive Review of Pathogenesis, Diagnosis and Treatment
Source: Cancers (Basel). 2023 Oct 6;15(19):4869. doi: 10.3390/cancers15194869 (PMC10571999; doi:10.3390/cancers15194869)
Supplement: Supplementary file 1 [file cancers-15-04869-s001.zip › cancers-2499097-supplementary.pdf]

Decoding Diffuse Midline Gliomas: A Comprehensive Review of Pathogenesis, Diagnosis, and Treatment (Supplementary Material)

Sarah Al Sharie, Dima Abu Laban and Maysa Al-Hussaini

Table S1. All currently available clinical trials examining DMG and DIPG.

NCT Number	Title	Status	Phases	Study Type	Interventions	Number of enrolled patients	Age	Location	Start Date	Completion Date	Publication
NCT02420613	Vorinostat and Temsirolimus With or Without Radiation Therapy in Treating Younger Patients With Newly Diagnosed or Progressive Diffuse Intrinsic Pontine Glioma	Active, not recruiting	Phase 1	Interventional	Drug: Temsirolimus, Vorinostat<br>Radiation: Radiation Therapy	18	7 Months - 21 Years	USA	October 5, 2015	October 31, 2023	[1]
NCT02525692	Oral ONC201 in Recurrent GBM, H3 K27M Glioma, and Midline Glioma	Active, not recruiting	Phase 2	Interventional	Drug: ONC201	89	≥ 16 Years	USA	January 2016	December 2023	[2]
NCT02717455	Trial of Panobinostat in Children With Diffuse Intrinsic Pontine Glioma	Active, not recruiting	Phase 1	Interventional	Drug: LBH589	53	2 - 21 Years	USA	June 28, 2016	March 31, 2024	N/A
NCT02960230	H3.3K27M Peptide Vaccine With Nivolumab for Children With Newly Diagnosed DIPG and Other Gliomas	Active, not recruiting	Phase 1/ Phase 2	Interventional	Biological: K27M peptide<br>Drug: Nivolumab	50	3 - 21 Years	USA	November 18, 2016	November 30, 2024	[3]
NCT03033992	Optune for Children With High-Grade Glioma or Ependymoma, and Optune With Radiation Therapy for Children With DIPG	Active, not recruiting	-	Interventional	Device: Optune System (NovoTTF-200A System, Tumor Treating Fields, TTFields)<br>Combination Product: Concurrent Optune and RT followed by Optune System alone	80	3 - 21 Years	USA	April 4, 2017	August 28, 2029	[4]
NCT03257631	A Study of Pomalidomide Monotherapy for Children and Young Adults With Recurrent or Progressive Primary Brain Tumors	Active, not recruiting	Phase 2	Interventional	Drug: Pomalidomide	53	1 Year - 21 Years	USA	August 22, 2017	September 27, 2023	[5]
NCT03389802	Phase I Study of APX005M in Pediatric CNS Tumors	Active, not recruiting	Phase 1	Interventional	Biological: APX005M treatment for newly diagnosed DIPG patients	32	1 Year - 21 Years	USA	March 1, 2018	March 31, 2024	N/A
NCT03416530	ONC201 in Pediatric H3 K27M Gliomas	Active, not recruiting	Phase 1	Interventional	Drug: ONC201	130	2 - 18 Years	USA	January 29, 2018	May 31, 2023	[6]
NCT03478462	Dose Escalation Study of CLR 131 in Children, Adolescents, and Young Adults With Relapsed or Refractory Malignant Tumors Including But	Active, not recruiting	Phase 1	Interventional	Drug: CLR 131	30	2 - 25 Years	USA	April 30, 2019	December 2024	[7]

	Not Limited to Neuroblastoma, Rhabdomyosarcoma, Ewings Sarcoma, and Osteosarcoma										
NCT03620032	Study of Re-irradiation at Relapse Versus RT and Multiple Elective rt Courses	Active, not recruiting	Phase 2	Interventional	Drug: Nimotuzumab, Vinorelbine<br>Other: Radiotherapy	54	2 - 21 Years	Italy	November 2, 2015	November 2, 2024	[8]
NCT03739372	Clinical Benefit of Using Molecular Profiling to Determine an Individualized Treatment Plan for Patients With High Grade Glioma	Active, not recruiting	-	Interventional	Other: Specialized tumor board recommendation	44	< 21 Years	USA	November 28, 2018	January 1, 2027	[9]
NCT03893487	Fimepinostat in Treating Brain Tumors in Children and Young Adults	Active, not recruiting	Early Phase 1	Interventional	Drug: Fimepinostat<br>Procedure: Therapeutic Conventional Surgery	30	3 - 39 Years	USA	August 7, 2019	December 1, 2027	N/A
NCT04239092	9-ING-41 in Pediatric Patients With Refractory Malignancies.	Active, not recruiting	Phase 1	Interventional	Drug: 9-ING-41, Irinotecan, Temozolomide, Cyclophosphamide, Topotecan	68	< 22 Years	USA	June 5, 2020	December 2024	[10]
NCT04295759	INCB7839 in Treating Children With Recurrent/Progressive High-Grade Gliomas	Active, not recruiting	Phase 1	Interventional	Drug: INCB7839	13	3 - 21 Years	USA	July 27, 2020	June 30, 2024	N/A
NCT04341311	Phase I Study of Marizomib + Panobinostat for Children With DIPG	Active, not recruiting	Phase 1	Interventional	Drug: Marizomib, Panobinostat	45	< 21 Years	USA	August 10, 2020	December 31, 2024	N/A
NCT04804709	Non-Invasive Focused Ultrasound (FUS) With Oral Panobinostat in Children With Progressive Diffuse Midline Glioma (DMG)	Active, not recruiting	Phase 1	Interventional	Drug: Panobinostat 15 MG<br>Device: Focused Ultrasound with neuro-navigator-controlled sonication	3	4 - 21 Years	USA	July 28, 2021	December 2025	N/A
NCT04870944	CBL0137 for the Treatment of Relapsed or Refractory Solid Tumors, Including CNS Tumors and Lymphoma	Active, not recruiting	Phase 1/Phase 2	Interventional	Drug: FACT Complex-targeting Curaxin CBL0137	38	12 Months - 30 Years	USA	January 18, 2022	December 31, 2026	N/A
NCT04911621	Adjuvant Dendritic Cell Immunotherapy for Pediatric Patients With High-grade Glioma or Diffuse Intrinsic Pontine Glioma	Active, not recruiting	Phase 1/Phase 2	Interventional	Biological: Dendritic cell vaccination + temozolomide-based chemoradiation +- conventional next-line treatment	10	12 Months - 17 Years	Belgium	September 10, 2021	June 1, 2027	N/A
NCT05518838	Expanded Access to OKN-007 for Patients With Diffuse Midline Glioma, H3 K27-altered	Available	-	Expanded Access:Intermediate-size Population	Drug: OKN-007	-	13 Months - 18 Years	USA	-	-	N/A
NCT00028795	Chemotherapy and Radiation Therapy After Surgery in Treating Children With Newly Diagnosed Astrocytoma, Glioblastoma Multiforme, Gliosarcoma, or Diffuse Intrinsic Pontine Glioma	Completed	Phase 2	Interventional	Drug: temozolomide<br>Radiation therapy	170	3-21 Years	USA	December 2002	March 2012	N/A
NCT00036569	A Phase II Study of Pegylated Interferon Alfa 2b (PEG-Intron(Trademark)) in Children With Diffuse Pontine Gliomas	Completed	Phase 2	Interventional	Procedure: adjuvant therapy<br>Biological: pegylated interferon alfa	32	< 21 Years	USA	May 2002	January 2012	N/A

NCT00561691	Nimotuzumab in Children With Intrinsic Pontine Glioma	Completed	-	Observational	-	41	3 - 20 Years	Germany	April 2006	January 2012	N/A
NCT00600054	Phase 2 Study of Nimotuzumab in Pediatric Recurrent Diffuse Intrinsic Pontine Glioma	Completed	Phase 2	Interventional	Drug: nimotuzumab	44	3 - 18 Years	USA	October 2007	December 2010	N/A
NCT00879437	Valproic Acid, Radiation, and Bevacizumab in Children With High Grade Gliomas or Diffuse Intrinsic Pontine Glioma	Completed	Phase 2	Interventional	Drug: Valproic acid, Bevacizumab<br>Radiation: Radiation therapy	38	3 - 21 Years	USA	September 1, 2009	October 2, 2017	[11]
NCT00890786	A Study of Bevacizumab Therapy in Patients With Newly Diagnosed High-Grade Gliomas and Diffuse Intrinsic Pontine Gliomas	Completed	Early Phase 1	Interventional	Drug: Temozolomide, Bevacizumab, Irinotecan	27	3 - 30 Years	USA	May 2009	December 2017	N/A
NCT00996723	Clinical Trial Evaluating the Combination of Vandetanib and Dasatinib During and After Radiation Therapy (RT) in Children With Newly Diagnosed Diffuse Intrinsic Pontine Glioma (DIPG)	Completed	Phase 1	Interventional	Drug: Vandetanib, Dasatinib	25	18 Months - 21 Years	USA	October 2009	June 2014	N/A
NCT01165333	Cilengitide in Combination With Irradiation in Children With Diffuse Intrinsic Pontine Glioma	Completed	Phase 1	Interventional	Drug: Cilengitide<br>Radiation: Concomitant radiotherapy<br>Biological: Pharmacokinetic, Pharmacogenetic and Exploratory investigation	32	6 Months - 21 Years	France	August 2010	March 2015	N/A
NCT01189266	Vorinostat and Radiation Therapy Followed by Maintenance Therapy With Vorinostat in Treating Younger Patients With Newly Diagnosed Diffuse Intrinsic Pontine Glioma	Completed	Phase 1/Phase 2	Interventional	Drug: Vorinostat<br>Radiation: 3-Dimensional Conformal Radiation Therapy, Intensity-Modulated Radiation Therapy<br>Other: Laboratory Biomarker Analysis	79	37 Months - 21 Years	USA	August 9, 2010	September 30, 2021	[12]
NCT01222754	Lenalidomide and Radiation Therapy in High Grade Gliomas or Diffuse Intrinsic Pontine Gliomas	Completed	Phase 1	Interventional	Drug: Lenalidomide<br>Radiation: XRT, 54-59.4 Gy	29	1 Year - 21 Years	USA	November 23, 2010	April 17, 2019	[13]
NCT01393912	PDGFR Inhibitor Crenolanib in Children/Young Adults With Diffuse Intrinsic Pontine Glioma or Recurrent High-Grade Glioma	Completed	Phase 1	Interventional	Drug: Crenolanib	55	18 Months - 21 Years	USA	July 2011	October 2016	[14]
NCT01445288	Exploratory Study of Effects of Radiation Therapy in Pediatric Patients With Central Nervous System Tumors	Completed	-	Observational	-	76	1 Month - 21 Years	USA	December 5, 2006	-	N/A
NCT01469247	Diffuse Intrinsic Pontine Glioma (DIPG) Reirradiation (ReRT)	Completed	Phase 1/Phase 2	Interventional	Radiation: Radiation Therapy	12	< 17 Years	USA	December 7, 2011	September 20, 2022	[15]
NCT01502917	Convection-Enhanced Delivery of 124I-Omburtamab for Patients With Non-Progressive	Completed	Phase 1	Interventional	Drug: Radioactive iodine-labeled monoclonal antibody	50	2 - 21 Years	USA	December 2011	January 2022	[16]

	Diffuse Pontine Gliomas Previously Treated With External Beam Radiation Therapy				omburtamab<br>Radiation: External Beam Radiotherapy						
NCT01514201	Veliparib, Radiation Therapy, and Temozolomide in Treating Younger Patients With Newly Diagnosed Diffuse Pontine Gliomas	Completed	Phase 1/ Phase 2	Interventional	Drug: Temozolomide, Veliparib<br>Radiation: 3-Dimensional Conformal Radiation Therapy, Intensity-Modulated Radiation Therapy<br>Other: Laboratory Biomarker Analysis, Pharmacological Study	66	< 21 Years	USA	February 1, 2012	March 28, 2018	[17]
NCT01635140	Hypofractionated Radiotherapy Versus Conventional Radiotherapy in Diffuse Brainstem Glioma in Children	Completed	Phase 3	Interventional	Radiation: Hypofractionated radiotherapy, Conventional arm	64	3 - 18 Years	Egypt	May 2010	July 2011	[18]
NCT01644773	Study of the Combination of Crizotinib and Dasatinib in Pediatric Research Participants With Diffuse Pontine Glioma (DIPG) and High-Grade Glioma (HGG)	Completed	Phase 1	Interventional	Drug: Crizotinib, Dasatinib	36	2 - 21 Years	USA	November 27, 2012	September 28, 2018	[19]
NCT01688401	Intra-arterial Chemotherapy for the Treatment of Progressive Diffuse Intrinsic Pontine Gliomas (DIPG).	Completed	Phase 1	Interventional	Drug: Melphalan hydrochloride	3	1 Month - 17 Years	USA	March 8, 2013	November 26, 2018	N/A
NCT01777633	Palliative Re-irradiation for Progressive Diffuse Intrinsic Pontine Glioma (DIPG) in Children	Completed	Phase 1/Phase 2	Interventional	Radiation: Palliative re-irradiation for progressive DIPG in children	15	1 Year - 22 Years	Israel	February 2013	February 2015	N/A
NCT01878266	Prospective Trial of Two Hypofractionated Radiotherapy Regimens Versus Conventional Radiotherapy in Diffuse Brainstem Glioma in Children	Completed	-	Interventional	Radiation: Hypofractionated Arm, Conventional Arm	119	2 - 18 Years	Egypt	February 2013	May 2016	[20]
NCT01922076	Adavosertib and Local Radiation Therapy in Treating Children With Newly Diagnosed Diffuse Intrinsic Pontine Gliomas	Completed	Phase 1	Interventional	Drug: Adavosertib<br>Radiation: Radiation Therapy<br>Other: Laboratory Biomarker Analysis, Pharmacological Study	46	37 Months - 21 Years	USA	September 3, 2013	September 30, 2022	N/A
NCT02151227	Magnesium and Metabolic Syndrome: A Dose-response Meta-analysis	Completed	-	Observational	-	12	18 - 90 Years	Republic of Korea	June 2014	December 2014	[21]
NCT02274987	Molecular Profiling for Individualized Treatment Plan for DIPG	Completed	-	Interventional	Radiation: Standard radiation therapy	38	< 25 Years	USA	September 16, 2014	February 24, 2019	
NCT02343406	Adult Study: ABT-414 Alone or ABT-414 Plus Temozolomide vs. Lomustine or Temozolomide for Recurrent Glioblastoma Pediatric Study: Evaluation of ABT-414 in Children With High Grade Gliomas	Completed	Phase 2	Interventional	Drug: Depatuxizumab mafodotin, Temozolomide, Lomustine	266	< 99 Years	USA	February 17, 2015	June 24, 2019	[22]
NCT02444546	Wild-Type Reovirus in Combination With Sargramostim in Treating Younger Patients With	Completed	Phase 1	Interventional	Biological: Sargramostim, Wild-type Reovirus	6	10 - 21 Years	USA	June 21, 2015	November 21, 2022	[23]

	High-Grade Relapsed or Refractory Brain Tumors				Other: Laboratory Biomarker Analysis						
NCT02502708	Study of the IDO Pathway Inhibitor, Indoximod, and Temozolomide for Pediatric Patients With Progressive Primary Malignant Brain Tumors	Completed	Phase 1	Interventional	Drug: Indoximod, Temozolomide, Cyclophosphamide, Etoposide<br>Radiation: Conformal Radiation	81	3 - 21 Years	USA	October 2015	February 28, 2020	[24]
NCT02644291	Phase I Study of Mebendazole Therapy for Recurrent/Progressive Pediatric Brain Tumors	Completed	Phase 1	Interventional	Drug: Mebendazole	16	1 Year - 21 Years	USA	May 2016	June 9, 2022	N/A
NCT02750891	A Study of DSP-7888 in Pediatric Patients With Relapsed or Refractory High Grade Gliomas	Completed	Phase 1/Phase 2	Interventional	Drug: DSP-7888	18	< 19 Years	Japan	April 2016	January 2020	N/A
NCT03086616	CED With Irinotecan Liposome Injection Using Real Time Imaging in Children With Diffuse Intrinsic Pontine Glioma (DIPG) (PNOC 009)	Completed	Phase 1	Interventional	Drug: Convection Enhanced Delivery (CED) of Nanoliposomal irinotecan (nal-IRI)	6	2 Years and older	USA	October 31, 2017	October 4, 2021	N/A
NCT03178032	Oncolytic Adenovirus, DNX-2401, for Naive Diffuse Intrinsic Pontine Gliomas	Completed	Phase 1	Interventional	Biological: DNX-2401	12	1 Year - 18 Years	Spain	May 26, 2017	January 31, 2021	[25]
NCT03355794	A Study of Ribociclib and Everolimus Following Radiation Therapy in Children With Newly Diagnosed Non-biopsied Diffuse Pontine Gliomas (DIPG) and RB+ Biopsied DIPG and High Grade Gliomas (HGG)	Completed	Phase 1	Interventional	Drug: Ribociclib, Everolimus	19	12 Months - 30 Years	USA	November 14, 2017	July 31, 2022	N/A
NCT03387020	Ribociclib and Everolimus in Treating Children With Recurrent or Refractory Malignant Brain Tumors	Completed	Phase 1	Interventional	Drug: Everolimus, Ribociclib<br>Other: Laboratory Biomarker Analysis, Pharmacological Study	22	1 Year - 21 Years	USA	January 13, 2018	April 1, 2020	N/A
NCT03566199	MTX110 by Convection-Enhanced Delivery in Treating Participants With Newly-Diagnosed Diffuse Intrinsic Pontine Glioma	Completed	Phase 1/Phase 2	Interventional	Drug: Panobinostat Nanoparticle Formulation MTX110, Convection-Enhanced Delivery (CED)	7	2 - 21 Years	USA	May 22, 2018	March 31, 2021	N/A
NCT03696355	Study of GDC-0084 in Pediatric Patients With Newly Diagnosed Diffuse Intrinsic Pontine Glioma or Diffuse Midline Gliomas	Completed	Phase 1	Interventional	Drug: GDC-0084<br>Radiation: radiation therapy	27	2 - 21 Years	USA	November 19, 2018	September 4, 2022	N/A
NCT03841435	Hypofractionated Radiotherapy for Recurrent DIPG	Enrolling by invitation	-	Interventional	Radiation: Hypofractionated Radiotherapy	6	< 30 Years	USA	January 31, 2018	July 31, 2023	N/A
NCT05392374	Expanded Access Use of ONC201 in a Patient With Diffuse Intrinsic Pontine Gliomas	No longer available	-	Expanded Access:Intermediate-size Population	Drug: ONC201	-	6 Months - 18 Years	USA	-	-	N/A
NCT05063357	131I-omburtamab Delivered by Convection-Enhanced Delivery in Patients With Diffuse Intrinsic Pontine Glioma	Not yet recruiting	Phase 1	Interventional	Drug: 131I-Omburtamab<br>Device: Convention Enhanced Delivery	36	3 - 21 Years	-	March 2023	January 31, 2027	N/A
NCT05096481	PEP-CMV Vaccine Targeting CMV Antigen to Treat Newly Diagnosed Pediatric HGG and DIPG and Recurrent Medulloblastoma	Not yet recruiting	Phase 2	Interventional	Drug: Temozolomide<br>Biological: Tetanus Diphtheria Vaccine, PEP-CMV	120	3 - 25 Years	USA	May 15, 2023	May 15, 2029	N/A

NCT05298995	GD2-CAR T Cells for Pediatric Brain Tumours	Not yet recruiting	Phase 1	Interventional	Biological: GD2-CART01 (iC9-GD2-CAR T-cells)	54	6 Months - 30 Years	-	May 2022	May 2037	N/A
NCT05478837	Genetically Modified Cells (KIND T Cells) for the Treatment of HLA-A*0201-Positive Patients With H3.3K27M-Mutated Glioma	Not yet recruiting	Phase 1	Interventional	Drug: Cyclophosphamide丨Drug: Fludarabine丨Biological: Autologous Anti-H3.3K27M TCR-expressing T-cells	12	3 - 21 Years	USA	May 30, 2023	December 31, 2028	N/A
NCT05544526	CAR T Cells to Target GD2 for DMG	Not yet recruiting	Phase 1	Interventional	Biological: GD2 CAR T cells	12	< 16 Years	-	May 30, 2023	December 2039	N/A
NCT05768880	Study of B7-H3, EGFR806, HER2, And IL13-Zetakine (Quad) CAR T Cell Locoregional Immunotherapy For Pediatric Diffuse Intrinsic Pontine Glioma, Diffuse Midline Glioma, And Recurrent Or Refractory Central Nervous System Tumors	Not yet recruiting	Phase 1	Interventional	Biological: SC-CAR4BRAIN	72	1 Year - 26 Years	USA	April 25, 2023	December 31, 2043	N/A
NCT05839379	Targeted Pediatric High-Grade Glioma Therapy	Not yet recruiting	-	Observational	-	450	12 Months - 30 Years	USA	August 15, 2023	August 15, 2033	N/A
NCT05843253	Study of Ribociclib and Everolimus in HGG and DIPG	Not yet recruiting	Phase 2	Interventional	Drug: Ribociclib, Everolimus	100	12 Months - 30 Years	USA	August 15, 2023	August 15, 2033	N/A
NCT01106794	Molecular Analysis of Samples From Patients With Diffuse Intrinsic Pontine Glioma and Brainstem Glioma	Recruiting	-	Observational	-	100	< 21 Years	USA	April 2010	April 2030	N/A
NCT01793168	Rare Disease Patient Registry & Natural History Study - Coordination of Rare Diseases at Sanford	Recruiting	-	Observational	-	20000	-	USA	July 2010	December 2100	N/A
NCT01837862	A Phase I Study of Mebendazole for the Treatment of Pediatric Gliomas	Recruiting	Phase 1/Phase 2	Interventional	Drug: Mebendazole, Vincristine, Carboplatin, Temozolomide, Bevacizumab Irinotecan	36	1 Year - 21 Years	USA	October 22, 2013	April 2025	N/A
NCT02359565	Pembrolizumab in Treating Younger Patients With Recurrent, Progressive, or Refractory High-Grade Gliomas, Diffuse Intrinsic Pontine Gliomas, Hypermutated Brain Tumors, Ependymoma or Medulloblastoma	Recruiting	Phase 1	Interventional	Biological: Pembrolizumab Procedure: Biospecimen Collection, Conventional Magnetic Resonance Imaging, Diffusion Tensor Imaging, Diffusion Weighted Imaging, Dynamic Contrast-enhanced MR Perfusion, Dynamic Susceptibility Contrast-Perfusion-Weighted Imaging, Magnetic Resonance Spectroscopic Imaging	110	1 Year - 30 Years	USA	May 22, 2015	December 31, 2026	N/A
NCT02644460	Abemaciclib in Children With DIPG or Recurrent/Refractory Solid Tumors	Recruiting	Phase 1	Interventional	Drug: Abemaciclib	60	2 - 25 Years	USA	February 2016	December 2024	N/A
NCT02992015	Gemcitabine in Newly-Diagnosed Diffuse Intrinsic Pontine Glioma	Recruiting	Early Phase 1	Interventional	Drug: Gemcitabine	10	3 - 17 Years	USA	September 23, 2016	December 2025	N/A

NCT03101813	International Diffuse Intrinsic Pontine Glioma (DIPG)/Diffuse Midline Glioma (DMG) Registry and Repository	Recruiting	-	Observational	-	1500	-	USA	June 2012	June 2047	N/A
NCT03126266	Re-Irradiation of Progressive or Recurrent DIPG	Recruiting	-	Interventional	Radiation: re-irradiation	27	-	Australia, Canada, New Zealand	April 15, 2017	December 2024	N/A
NCT03243461	International Cooperative Phase III Trial of the HIT-HGG Study Group (HIT-HGG-2013)	Recruiting	Phase 3	Interventional	Drug: Temozolomide, Valproic Acid	167	3 - 17 Years	Germany	July 17, 2018	December 31, 2023	N/A
NCT03396575	Brain Stem Gliomas Treated With Adoptive Cellular Therapy During Focal Radiotherapy Recovery Alone or With Dose-intensified Temozolomide (Phase I)	Recruiting	Phase 1	Interventional	Drug: Cyclophosphamide + Fludarabine Lymphodepletive Conditioning, Dose-Intensified TMZ, Td vaccine<br>Biological: TTRNA-DC vaccines with GM-CSF, TTRNA-xALT, Autologous Hematopoietic Stem Cells (HSC)	21	3 - 30 Years	USA	May 17, 2018	June 2025	N/A
NCT03598244	Volitinib in Treating Patients With Recurrent or Refractory Primary CNS Tumors	Recruiting	Phase 1	Interventional	Drug: Savolitinib	50	6 - 21 Years	USA	October 15, 2018	January 31, 2025	N/A
NCT03605550	A Phase 1b Study of PTC596 in Children With Newly Diagnosed Diffuse Intrinsic Pontine Glioma and High Grade Glioma	Recruiting	Phase 1	Interventional	Drug: PTC596<br>Radiation: Radiotherapy	64	12 Months - 21 Years	USA	August 1, 2018	July 1, 2028	N/A
NCT03690869	REGN2810 in Pediatric Patients With Relapsed, Refractory Solid, or Central Nervous System (CNS) Tumors and Safety and Efficacy of REGN2810 in Combination With Radiotherapy in Pediatric Patients With Newly Diagnosed or Recurrent Glioma	Recruiting	Phase 1/Phase 2	Interventional	Drug: cemiplimab (monotherapy), cemiplimab (maintenance)<br>Radiation: Conventional or hypofractionated, Re-irradiation	130	< 25 Years	USA	September 24, 2018	November 20, 2024	N/A
NCT03709680	Study Of Palbociclib Combined With Chemotherapy In Pediatric Patients With Recurrent/Refractory Solid Tumors	Recruiting	Phase 1/Phase 2	Interventional	Drug: Palbociclib, Temozolomide, Irinotecan, Topotecan, Cyclophosphamide	184	2 - 20 Years	USA	May 24, 2019	March 15, 2025	[26]
NCT04049669	Pediatric Trial of Indoximod With Chemotherapy and Radiation for Relapsed Brain Tumors or Newly Diagnosed DIPG	Recruiting	Phase 2	Interventional	Drug: Indoximod, Temozolomide, Cyclophosphamide Etoposide, Lomustine<br>Radiation: Partial Radiation, Full-dose Radiation	140	3 - 21 Years	USA	October 2, 2019	October 2, 2027	N/A
NCT04099797	C7R-GD2.CAR T Cells for Patients With GD2-expressing Brain Tumors (GAIL-B)	Recruiting	Phase 1	Interventional	Genetic: (C7R)-GD2.CART cells	34	12 Months - 21 Years	USA	February 3, 2020	February 2039	N/A
NCT04185038	Study of B7-H3-Specific CAR T Cell Locoregional Immunotherapy for Diffuse Intrinsic Pontine Glioma/Diffuse Midline Glioma and	Recruiting	Phase 1	Interventional	Biological: SCRI-CARB7H3(s); B7H3-specific chimeric antigen receptor (CAR) T cel	90	1 Year - 26 Years	USA	December 11, 2019	May 2041	N/A

	Recurrent or Refractory Pediatric Central Nervous System Tumors										
NCT04196413	GD2 CAR T Cells in Diffuse Intrinsic Pontine Gliomas(DIPG) & Spinal Diffuse Midline Glioma(DMG)	Recruiting	Phase 1	Interventional	Drug: GD2 CAR T cells, Fludarabine, Cyclophosphamide	54	2 - 30 Years	USA	June 4, 2020	July 31, 2042	[27]
NCT04238819	A Study of Abemaciclib (LY2835219) in Combination With Other Anti-Cancer Treatments in Children and Young Adult Participants With Solid Tumors, Including Neuroblastoma	Recruiting	Phase 1/Phase 2	Interventional	Drug: Abemaciclib, Irinotecan, Temozolomide, Dinutuximab, GM-CSF	117	< 21 Years	USA	November 9, 2020	December 21, 2028	[28]
NCT04250064	A Study of Low Dose Bevacizumab With Conventional Radiotherapy Alone in Diffuse Intrinsic Pontine Glioma	Recruiting	Phase 2	Interventional	Drug: Bevacizumab Injection Radiation: Ultra-low-dose RT	40	3 - 18 Years	India	February 4, 2020	February 2023	N/A
NCT04264143	CED of MTX110 Newly Diagnosed Diffuse Midline Gliomas	Recruiting	Phase 1	Interventional	Drug: Infusate with MTX110 and gadolinium丨Device: Convection-Enhanced Delivery (CED)	9	3 - 18 Years	USA	March 10, 2020	February 2024	N/A
NCT04541082	Phase I Study of Oral ONC206 in Recurrent and Rare Primary Central Nervous System Neoplasms	Recruiting	Phase 1	Interventional	Drug: ONC206	102	≥ 18 Years	USA	October 26, 2020	February 2025	[29]
NCT04655404	A Pilot Study of Larotrectinib for Newly-Diagnosed High-Grade Glioma With NTRK Fusion	Recruiting	Early Phase 1	Interventional	Drug: Larotrectinib Procedure: Larotrectinib surgical	15	< 21 Years	USA	April 8, 2021	December 1, 2025	N/A
NCT04670016	HRQL and Symptom Assessment for Patients With DIPG or Recurrent and Re-irradiated Brain Tumours and Their Caregivers	Recruiting	-	Observational	-	57	2 - 21 Years	Canada	July 2, 2020	December 2023	N/A
NCT04732065	ONC206 for Treatment of Newly Diagnosed, or Recurrent Diffuse Midline Gliomas, and Other Recurrent Malignant CNS Tumors (PNOC 023)	Recruiting	Phase 1	Interventional	Drug: ONC206 Radiation: Standard of Care Radiation Therapy	256	2 - 21 Years	USA	August 23, 2021	December 31, 2025	N/A
NCT04749641	Neoantigen Vaccine Therapy Against H3.3-K27M Diffuse Intrinsic Pontine Glioma	Recruiting	Phase 1	Interventional	Biological: Histone H3.3-K27M Neoantigen Vaccine Therapy	30	≥ 5 Years	China	March 8, 2021	April 1, 2024	[30]
NCT04758533	Clinical Trial to Assess the Safety and Efficacy of AloCELYVIR With Newly Diagnosed Diffuse Intrinsic Pontine Glioma (DIPG) in Combination With Radiotherapy or Medulloblastoma in Monotherapy	Recruiting	Phase 1/Phase 2	Interventional	Biological: AloCELYVIR	12	1 Year - 21 Years	Spain	April 19, 2021	October 2024	N/A
NCT04771897	A Study of BXQ-350 in Children With Newly Diagnosed Diffuse Intrinsic Pontine Glioma (DIPG) or Diffuse Midline Glioma (DMG)	Recruiting	Phase 1	Interventional	Drug: BXQ-350 - Part 1 Dose Escalation: Safety and Tolerance, BXQ-350 - Part 2 BXQ-350 Tumor and Plasma Concentrations	22	1 Year - 30 Years	USA	May 24, 2021	December 2024	N/A
NCT04808245	A MultIceNTER Phase I Peptide VaCcine Trial for the Treatment of H3-Mutated Gliomas	Recruiting	Phase 1	Interventional	Drug: Tecentriq 1200 MG in 20 ML Injection Biological: H3K27M peptide vaccine Other: Imiquimod (5%)	15	≥ 18 Years	Germany	February 15, 2023	March 2025	N/A

NCT04837547	PEACH TRIAL- Precision Medicine and Adoptive Cellular Therapy	Recruiting	Phase 1	Interventional	Biological: Tumor-specific ex vivo expanded autologous lymphocyte transfer (TTRNA-xALT)	24	1 Year - 30 Years	USA	September 20, 2021	September 2030	N/A
NCT04908176	A Drug-drug Interaction Study of Avapritinib and Midazolam	Recruiting	Phase 1	Interventional	Drug: Avapritinib, Midazolam	20	18 Years and older	USA	August 24, 2022	December 31, 2023	N/A
NCT04943848	rHSC-DIPGVax Plus Checkpoint Blockade for the Treatment of Newly Diagnosed DIPG and DMG	Recruiting	Phase 1	Interventional	Biological: rHSC-DIPGVax<br>Drug: Balstilimab, Zalifrelimab	36	12 Months - 18 Years	USA	January 10, 2022	March 2025	N/A
NCT04978727	A Pilot Study of SurVaxM in Children Progressive or Relapsed Medulloblastoma, High Grade Glioma, Ependymoma and Newly Diagnosed Diffuse Intrinsic Pontine Glioma	Recruiting	Phase 1	Interventional	Biological: SurVaxM for patients with non-relapsed DIPG post radiation-therapy ages ≥1 and ≤21 years	35	1 Year - 21 Years	USA	July 1, 2022	June 30, 2028	N/A
NCT05009992	Combination Therapy for the Treatment of Diffuse Midline Gliomas	Recruiting	Phase 2	Interventional	Drug: ONC201, Paxalisib<br>Radiation: Radiation Therapy	324	2 - 39 Years	USA	October 20, 2021	June 30, 2027	N/A
NCT05077735	Stereotactic Biopsy Split-Course Radiation Therapy in Diffuse Midline Glioma, SPORT-DMG Study	Recruiting	Phase 2	Interventional	Radiation: Hypofractionated Radiation Therapy<br>Other: Quality-of-Life Assessment, Questionnaire Administration	18	≥ 1 Year	USA	October 6, 2021	October 15, 2025	N/A
NCT05099003	A Study of the Drug Selinexor With Radiation Therapy in Patients With Newly-Diagnosed Diffuse Intrinsic Pontine (DIPG) Glioma and High-Grade Glioma (HGG)	Recruiting	Phase 1/Phase 2	Interventional	Drug: Selinexor<br>Procedure: Biopsy, Magnetic Resonance Imaging<br>Radiation: Radiation Therapy	210	12 Months - 21 Years	USA	May 5, 2022	June 30, 2024	N/A
NCT05123534	A Phase 1/2 Study of Sonodynamic Therapy Using SONALA-001 and Exablate 4000 Type 2 in Patients With DIPG	Recruiting	Phase 1/Phase 2	Interventional	Combination Product: SONALA-001 (ALA) and MR-Guided Focused Ultrasound device (MRgFUS)	27	≥ 5 Years	USA	August 12, 2022	August 12, 2025	[31]
NCT05259605	Observational Study for Assessing Treatment and Outcome of Patients With Primary Brain Tumours Using cIMPACT-NOW and 2021 WHO Classification	Recruiting	-	Observational	-	1250	≥ 18 Years	Spain	March 28, 2023	December 15, 2026	N/A
NCT05278208	Lutathera for Treatment of Recurrent or Progressive High-Grade CNS Tumors or Meningiomas Expressing SST2A	Recruiting	Phase 1/Phase 2	Interventional	Drug: LUTATHERA® (Lutetium Lu 177 dotatate)	65	≥ 4 Years	USA	November 21, 2022	November 2027	N/A
NCT05413304	Abemaciclib Neuropharmacokinetics of Diffuse Midline Glioma Using Intratumoral Microdialysis	Recruiting	Phase 1	Interventional	Drug: pre-operative abemaciclib, abemaciclib + temozolomide<br>Device: Device for Cerebral Fluid Dialysate Collection, Ashion Analytics GEM ExTra	7	18 - 39 Years	USA	April 7, 2023	August 31, 2024	N/A
NCT05476939	Biological Medicine for Diffuse Intrinsic Pontine Glioma (DIPG) Eradication 2.0	Recruiting	Phase 3	Interventional	Drug: Everolimus, ONC201<br>Radiation: Radiotherapy	368	≥ 6 Months	France	September 29, 2022	September 2031	N/A

NCT05500508	Oral AMXT 1501 Dicaprate in Combination With IV DFMO	Recruiting	Phase 1/Phase 2	Interventional	Drug: AMXT1501, DFMO	56	≥ 12 Years	USA	November 29, 2022	March 31, 2024	N/A
NCT05580562	ONC201 in H3 K27M-mutant Diffuse Glioma Following Radiotherapy (the ACTION Study)	Recruiting	Phase 3	Interventional	Drug: ONC201, ONC201 + Placebo<br>Other: Placebo	450	-	USA	January 23, 2023	August 2026	N/A
NCT05630209	Blood Brain Barrier (BBB) Disruption Using Exablate Focused Ultrasound With Doxorubicin for Treatment of Pediatric DIPG	Recruiting	Phase 1/Phase 2	Interventional	Drug: Doxorubicin<br>Device: Exablate	10	5 - 21 Years	USA	February 2023	January 2026	N/A
NCT05717712	Oncolytic Virus Ad-TD-nsIL12 for Primary Pediatric Diffuse Intrinsic Pontine Glioma	Recruiting	Phase 1	Interventional	Biological: Ad-TD-nsIL12	18	1 Year - 18 Years	China	January 4, 2023	January 4, 2028	N/A
NCT05762419	FUS Etoposide for DMG - A Feasibility Study	Recruiting	Phase 1	Interventional	Drug: Etoposide; Oral, 50 Mg<br>Device: Focused ultrasound with neuro-navigator-controlled sonication	10	4 - 21 Years	USA	March 2023	January 2027	N/A
NCT05835687	Loc3CAR: Locoregional Delivery of B7-H3-CAR T Cells for Pediatric Patients With Primary CNS Tumors	Recruiting	Phase 1	Interventional	Drug: B7-H3-CAR T cells	36	< 21 Years	USA	April 25, 2023	March 2028	N/A
NCT02742883	A Study of Atengenal and Astugenal in Diffuse, Intrinsic Pontine Glioma (DIPG)	Suspended	Phase 2	Interventional	Drug: Antineoplaston therapy (Atengenal + Astugenal)	1	3 Months - 99 Years	USA	April 13, 2016	March 2028	N/A
NCT01058850	Phase I Rindopepimut After Conventional Radiation in Children w/ Diffuse Intrinsic Pontine Gliomas	Terminated	Phase 1	Interventional	Biological: Rindopepimut	3	3 - 18 Years	USA	June 2011	September 2013	N/A
NCT01182350	Molecularly Determined Treatment of Diffuse Intrinsic Pontine Gliomas (DIPG)	Terminated	Phase 2	Interventional	Drug: Bevacizumab, Erlotinib, Temozolomide<br>Radiation: Radiation	53	3 - 18 Years	USA	September 2011	June 2016	N/A
NCT01400672	Imiquimod/Brain Tumor Initiating Cell (BTIC) Vaccine in Brain Stem Glioma	Terminated	Phase 1	Interventional	Drug: Imiquimod<br>Biological: Tumor Lysate Vaccine<br>Radiation: Radiation therapy	8	> 3 Years	USA	July 17, 2012	October 8, 2018	N/A
NCT01517776	Cilengitide and Metronomic Temozolomide for Relapsed or Refractory High Grade Gliomas or Diffuse Intrinsic Pontine Gliomas in Children and Adolescents	Terminated	Phase 2	Interventional	Drug: Cilengitide, Temozolomide	28	3 - 17 Years	Germany	January 2012	April 2014	N/A
NCT01774253	Erivedge (Vismodegib) in the Treatment of Pediatric Patients With Refractory Pontine Glioma	Terminated	Phase 2	Interventional	Drug: Vismodegib	9	3 - 18 Years	USA	May 2013	October 2015	N/A
NCT01836549	Imetelstat Sodium in Treating Younger Patients With Recurrent or Refractory Brain Tumors	Terminated	Phase 2	Interventional	Drug: imetelstat sodium	43	12 Months - 21 Years	USA	March 2013	April 2016	N/A

NCT01884740	Intraarterial Infusion Of Erbitux and Bevacizumab For Relapsed/Refractory Intracranial Glioma In Patients Under 22	Terminated	Phase 1/Phase 2	Interventional	Drug: SIACI of Erbitux and Bevacizumab	13	1 Year - 21 Years	USA	June 2013	October 2018	[32]
NCT02607124	A Phase I/II Study of Ribociclib,a CDK4/6 Inhibitor, Following Radiation Therapy	Terminated	Phase 1/Phase 2	Interventional	Drug: Ribociclib	11	12 Months - 30 Years	USA	April 2016	August 2018	N/A
NCT02758366	Prolonged Exposure to Doxorubicin in Patients With Glioblastoma Multiforme and Diffuse Intrinsic Pontine Glioma	Terminated	Phase 2	Interventional	Drug: Doxorubicin	21	3 - 30 Years	Italy	February 2016	January 16, 2020	N/A
NCT03330197	A Study of Ad-RTS-hIL-12 + Veledimex in Pediatric Subjects With Brain Tumors Including DIPG	Terminated	Phase 1/Phase 2	Interventional	Drug: Oral Veledimex - Arm 2 (DIPG)<br>Biological: Ad-RTS-hIL-12	6	0 - 21 Years	USA	September 26, 2017	September 10, 2021	N/A
NCT03967093	A Study of BXQ-350 in Children and Young Adults With Relapsed Solid Tumors	Terminated	Phase 1	Interventional	Drug: BXQ-350	9	1 Year - 30 Years	USA	April 15, 2019	January 17, 2020	[33]
NCT04730349	A Study of Bempegaldesleukin (BEMPEG: NKTR-214) in Combination With Nivolumab in Children, Adolescents and Young Adults With Recurrent or Treatment-resistant Cancer	Terminated	Phase 1/Phase 2	Interventional	Biological: Nivolumab, NKTR-214	15	< 30 Years	USA	June 3, 2021	June 22, 2022	N/A
NCT00278278	Combination Chemotherapy and Radiation Therapy With or Without Methotrexate in Treating Young Patients With Newly Diagnosed Gliomas	Unknown status	Phase 3	Interventional	Drug: Cisplatin, Etoposide, Ifosfamide, Lomustine, Methotrexate, Prednisone, Vincristine sulfate	150	3 - 18 Years	USA	September 2003	-	N/A
NCT01952769	Anti PD1 Antibody in Diffuse Intrinsic Pontine Glioma	Unknown status	Phase 1/Phase 2	Interventional	Biological: MDV9300	50	3 - 21 Years	Israel	February 2014	April 2019	N/A
NCT02233049	Biological Medicine for Diffuse Intrinsic Pontine Glioma (DIPG) Eradication	Unknown status	Phase 2	Interventional	Drug: Erlotinib, Everolimus, Dasatinib	250	6 Months - 25 Years	France	October 2014	October 2018	N/A
NCT02840123	Safety Study of DIPG Treatment With Autologous Dendritic Cells Pulsed With Lysated Allegenic Tumor Lines	Unknown status	Phase 1	Interventional	Biological: Autologous dendritic cells	10	3 - 18 Years	Spain	June 2016	March 2019	N/A
NCT03914768	Immune Modulatory DC Vaccine Against Brain Tumor	Unknown status	Phase 1	Interventional	Biological: Immunomodulatory DC vaccine to target DIPG and GBM	10	1 Year - 75 Years	China	March 31, 2019	December 31, 2022	N/A
NCT04532229	Nimotuzumab in Combined With Concurrent Radiochemotherapy in the Treatment of Newly Diagnosed Diffuse Intrinsic Pontine Glioma (DIPG) in Children	Unknown status	Phase 3	Interventional	Drug: Nimotuzumab+CRT(concurrent IMRT and TMZ)	48	3 - 15 Years	China	April 3, 2021	December 8, 2022	N/A
NCT03632317	A Study of Panobinostat in Combination With Everolimus for Children and Young Adults With Gliomas	Withdrawn	Phase 2	Interventional	Drug: Panobinostat, Everolimus	0	2 - 30 Years	USA	October 2019	October 2025	N/A

NCT04051047	Gemcitabine in Newly-Diagnosed Diffuse Midline Glioma	Withdrawn	Early Phase 1	Interventional	Drug: Gemcitabine<br>Procedure: Tumor biopsy and blood draw	0	3 - 17 Years	USA	November 30, 2019	December 2021	N/A

References

1. In Proceedings of theKhatua, S.; Chandra, J.; Gopalakrishnan, V.; Ketonen, L.; Johnson, M.; Sandberg, D.; Fuller, G.; Morrell, M.; Meador, H.; Bolton, K.; et al. *Pdct-16. A Phase I Study of Suberoylanilide Hydroxamic Acid (Saha) with Temsirolimus in Children with Newly Diagnosed or Progressive Diffuse Intrinsic Pontine Glioma (Dipg)*; Neuro Oncol. 2019 Nov;21(Suppl 6):vi186-7. doi: 10.1093/neuonc/noz175.776. Epub 2019 Nov 11.
2. Arrillaga-Romany, I.; Kurz, S.C.; Tarapore, R.; Sumrall, A.; Butowski, N.A.; Harrison, R.A.; Groot, J.F.D.; Chi, A.S.; Shonka, N.A.; Umemura, Y.; et al. Single-agent ONC201 in recurrent H3 K27M-mutant diffuse midline glioma. **2020**, *38*, 3615-3615, doi:10.1200/JCO.2020.38.15_suppl.3615.
3. Mueller, S.; Taitt, J.M.; Villanueva-Meyer, J.E.; Bonner, E.R.; Nejo, T.; Lulla, R.R.; Goldman, S.; Banerjee, A.; Chi, S.N.; Whipple, N.S.; et al. Mass cytometry detects H3.3K27M-specific vaccine responses in diffuse midline glioma. *The Journal of clinical investigation* **2020**, *130*, 6325-6337, doi:10.1172/jci140378.
4. Goldman, S.; Hwang, E.; Lai, J.S.; Kocak, M.; Lulla, R.; Dhall, G.; Robison, N.; Onar-Thomas, A.; I, J.D. *Pdct-07. Feasibility Trial of Ttfields (Tumor Treating Fields) for Children with Recurrent or Progressive Supratentorial High-Grade Glioma (Hgg) and Ependymoma: A Pediatric Brain Tumor Consortium Study: Pbtc-048*; Neuro Oncol. 2018 Nov;20(Suppl 6):vi201-2. doi: 10.1093/neuonc/noy148.837. Epub 2018 Nov 5.
5. Fangusaro, J.R.; Locatelli, F.; Garré, M.L.; Marshall, L.V.; Massimino, M.; Benettaib, B.; Biserna, N.; Poon, J.; Quan, J.; Conlin, E.; et al. A phase II clinical study of pomalidomide (CC-4047) monotherapy for children and young adults with recurrent or progressive primary brain tumors. **2019**, *37*, 10035-10035, doi:10.1200/JCO.2019.37.15_suppl.10035.
6. Gardner, S.L.; Allen, J.C.; Zaky, W.T.; Odia, Y.; Daghistani, D.; Khatib, Z.; Koschmann, C.J.; Aguilera, D.; MacDonald, T.J.; Chi, A.S.; et al. ONC201 in previously-irradiated pediatric H3 K27M-mutant glioma. **2019**, *37*, 10046-10046, doi:10.1200/JCO.2019.37.15_suppl.10046.
7. Puccetti, D.; Otto, M.; Morgenstern, D.; DeSantes, K.; Cho, S.; Hall, L.; Oliver, K.; Longcor, J. *Epct-09. Clr 131 in Patients with Relapsed or Refractory Pediatric Malignancies*; Neuro Oncol. 2020 Dec 4;22(Suppl 3):iii305. doi: 10.1093/neuonc/noaa222.133. eCollection 2020 Dec.
8. Iannó, M.F.; Biassoni, V.; Schiavello, E.; Carenzo, A.; Boschetti, L.; Gandola, L.; Diletto, B.; Marchesi, E.; Vegetti, C.; Molla, A.; et al. A microRNA Prognostic Signature in Patients with Diffuse Intrinsic Pontine Gliomas through Non-Invasive Liquid Biopsy. *Cancers* **2022**, *14*, doi:10.3390/cancers14174307.
9. Roberts, H.; Ravi, K.; Schepers, A.; Marini, B.; Kline, C.; Mueller, S.; Koschmann, C.; Franson, A. *Clrm-06. Comparison of Individualized Anti-Cancer Therapy Regimens Recommended by a Multidisciplinary Molecularly-Driven Tumor Board in a Pediatric Dipg Clinical Trial (Pnoc003) Versus Those Selected by the Cns-Tap Tool*; Neurooncol Adv. 2021 Sep 21;3(Suppl 4):iv2. doi: 10.1093/noajnl/vdab112.005. eCollection 2021 Oct.
10. DeNardo, B.; Foster, J.; Pinto, N.R.; Sholler, G.L.S.; Vo, K.T.; Desai, A.V.; Sun, J.; Wagner, L.M.; Macy, M.E.; Mody, R.; et al. Phase 1/2 study of elraglusib (9-ING-41), a small molecule selective glycogen synthase kinase-3 beta (GSK-3β) inhibitor, alone or with irinotecan, temozolomide/irinotecan or cyclophosphamide/topotecan in pediatric patients with refractory malignancies: Interim results. **2022**, *40*, e22015-e22015, doi:10.1200/JCO.2022.40.16_suppl.e22015.
11. Su, J.M.; Murray, J.C.; McNall-Knapp, R.Y.; Bowers, D.C.; Shah, S.; Adesina, A.M.; Paulino, A.C.; Jo, E.; Mo, Q.; Baxter, P.A.; et al. A phase 2 study of valproic acid and radiation, followed by maintenance valproic acid and bevacizumab in children with newly diagnosed diffuse intrinsic pontine glioma or high-grade glioma. *Pediatric blood & cancer* **2020**, *67*, e28283, doi:10.1002/pbc.28283.
12. Su, J.M.; Kilburn, L.B.; Mansur, D.B.; Krailo, M.; Buxton, A.; Adekunle, A.; Gajjar, A.; Adamson, P.C.; Weigel, B.; Fox, E.; et al. Phase I/II trial of vorinostat and radiation and maintenance vorinostat in children with diffuse intrinsic pontine glioma: A Children's Oncology Group report. *Neuro-oncology* **2022**, *24*, 655-664, doi:10.1093/neuonc/noab188.
13. Hipp, S.J.; Goldman, S.; Kaushal, A.; Krauze, A.; Citrin, D.; Glod, J.; Walker, K.; Shih, J.H.; Sethumadhavan, H.; O'Neill, K.; et al. A phase I trial of lenalidomide and radiotherapy in children with diffuse intrinsic pontine gliomas or high-grade gliomas. *Journal of neuro-oncology* **2020**, *149*, 437-445, doi:10.1007/s11060-020-03627-0.
14. Bisbee, C.; Campagne, O.; Gajjar, A.; Tinkle, C.L.; Stewart, C.F. Population pharmacokinetics of crenolanib in children and young adults with brain tumors. *Cancer chemotherapy and pharmacology* **2022**, *89*, 459-468, doi:10.1007/s00280-022-04412-8.
15. Bronk, J.K.; Hou, P.; Amsbaugh, M.J.; Khatua, S.; Mahajan, A.; Ketonen, L.; McGovern, S.L. Sequential Diffusion Tensor Imaging and Magnetic Resonance Spectroscopy in Patients Undergoing Reirradiation for Progressive Diffuse Intrinsic Pontine Glioma. *Advances in radiation oncology* **2022**, *7*, 100847, doi:10.1016/j.adro.2021.100847.
16. Bander, E.D.; Ramos, A.D.; Wembacher-Schroeder, E.; Ivasyk, I.; Thomson, R.; Morgenstern, P.F.; Souweidane, M.M. Repeat convection-enhanced delivery for diffuse intrinsic pontine glioma. *Journal of neurosurgery. Pediatrics* **2020**, *26*, 661-666, doi:10.3171/2020.6.Peds20280.
17. Baxter, P.A.; Su, J.M.; Onar-Thomas, A.; Billups, C.A.; Li, X.N.; Poussaint, T.Y.; Smith, E.R.; Thompson, P.; Adesina, A.; Ansell, P.; et al. A phase I/II study of veliparib (ABT-888) with radiation and temozolomide in newly diagnosed diffuse pontine glioma: a Pediatric Brain Tumor Consortium study. *Neuro-oncology* **2020**, *22*, 875-885, doi:10.1093/neuonc/noaa016.

18. Zaghloul, M.S.; Eldebawy, E.; Ahmed, S.; Mousa, A.G.; Amin, A.; Refaat, A.; Zaky, I.; Elkhateeb, N.; Sabry, M. Hypofractionated conformal radiotherapy for pediatric diffuse intrinsic pontine glioma (DIPG): a randomized controlled trial. *Radiotherapy and oncology : journal of the European Society for Therapeutic Radiology and Oncology* **2014**, *111*, 35-40, doi:10.1016/j.radonc.2014.01.013.
19. Gibson, E.G.; Campagne, O.; Selvo, N.S.; Gajjar, A.; Stewart, C.F. Population pharmacokinetic analysis of crizotinib in children with progressive/recurrent high-grade and diffuse intrinsic pontine gliomas. *Cancer chemotherapy and pharmacology* **2021**, *88*, 1009-1020, doi:10.1007/s00280-021-04357-4.
20. Park, J.; Yea, J.W.; Park, J.W. Hypofractionated radiotherapy versus conventional radiotherapy for diffuse intrinsic pontine glioma: A systematic review and meta-analysis. *Medicine* **2020**, *99*, e22721, doi:10.1097/md.0000000000022721.
21. La, S.A.; Lee, J.Y.; Kim, D.H.; Song, E.L.; Park, J.H.; Ju, S.Y. Low Magnesium Levels in Adults with Metabolic Syndrome: a Meta-Analysis. *Biological trace element research* **2016**, *170*, 33-42, doi:10.1007/s12011-015-0446-9.
22. Van Den Bent, M.; Eoli, M.; Sepulveda, J.M.; Smits, M.; Walenkamp, A.; Frenel, J.S.; Franceschi, E.; Clement, P.M.; Chinot, O.; De Vos, F.; et al. INTELLANCE 2/EORTC 1410 randomized phase II study of Depatux-M alone and with temozolomide vs temozolomide or lomustine in recurrent EGFR amplified glioblastoma. *Neuro-oncology* **2020**, *22*, 684-693, doi:10.1093/neuonc/noz222.
23. Schuelke, M.R.; Gundelach, J.H.; Coffey, M.; West, E.; Scott, K.; Johnson, D.R.; Samson, A.; Melcher, A.; Vile, R.G.; Bram, R.J. Phase I trial of sargramostim/pelareorep therapy in pediatric patients with recurrent or refractory high-grade brain tumors. *Neuro-Oncology Advances* **2022**, *4*, doi:10.1093/noajnl/vdac085.
24. S. Johnson, T.; Pacholczyk, R.; Aguilera, D.; Al-Basheer, A.; Bajaj, M.; Berrong, Z.; Castellino, R.C.; Eaton, B.R.; Esiashvili, N.; Foreman, N.; et al. IMMU-04. FIRST-IN-CHILDREN PHASE 1B STUDY USING THE IDO PATHWAY INHIBITOR INDOXIMOD IN COMBINATION WITH RADIATION AND CHEMOTHERAPY FOR CHILDREN WITH NEWLY DIAGNOSED DIPG (NCT02502708, NLG2105). *Neuro-oncology* **2021**, *23*, i27-i27, doi:10.1093/neuonc/noab090.112 %J Neuro-Oncology.
25. Gállego Pérez-Larraya, J.; Garcia-Moure, M.; Labiano, S.; Patiño-García, A.; Dobbs, J.; Gonzalez-Huarriz, M.; Zalacain, M.; Marrodan, L.; Martinez-Velez, N.; Puigdelloses, M.; et al. Oncolytic DNX-2401 Virus for Pediatric Diffuse Intrinsic Pontine Glioma. *The New England journal of medicine* **2022**, *386*, 2471-2481, doi:10.1056/NEJMoa2202028.
26. Laetsch, T.W.; Janeway, K.A.; Hawkins, D.S.; Juszczak-Kosela, K.; Muscal, J.A.; Collier, A.B. Phase 2 study to evaluate palbociclib in combination with irinotecan and temozolomide in pediatric patients with recurrent or refractory Ewing sarcoma. **2022**, *40*, TPS11583-TPS11583, doi:10.1200/JCO.2022.40.16_suppl.TPS11583.
27. Majzner, R.G.; Ramakrishna, S.; Yeom, K.W.; Patel, S.; Chinnasamy, H.; Schultz, L.M.; Richards, R.M.; Jiang, L.; Barsan, V.; Mancusi, R.; et al. GD2-CAR T cell therapy for H3K27M-mutated diffuse midline gliomas. *Nature* **2022**, *603*, 934-941, doi:10.1038/s41586-022-04489-4.
28. Bear, M.; DeNardo, B.; Federman, N.; Krystal, J.; Sholler, G.L.S.; Zhou, Y.; Knoderer, H.; Nabinger, S.C.; Hoffman, L.M. Phase 1b/2 study of abemaciclib combined with chemoimmunotherapy in pediatric and young adult patients with relapsed/refractory neuroblastoma (JPCS Part C). **2023**, *41*, TPS10068-TPS10068, doi:10.1200/JCO.2023.41.16_suppl.TPS10068.
29. Theeler, B.J.; Jung, J.; Burton, E.; Leeper, H.; Wu, J.; Zaghloul, K.; Ray-Chaudhury, A.; Quezado, M.; Raffeld, M.; Yuan, Y.; et al. First-in-human dose escalation and food effect study of oral ONC206 in adults with recurrent primary CNS neoplasms. **2021**, *39*, TPS2072-TPS2072, doi:10.1200/JCO.2021.39.15_suppl.TPS2072.
30. Ji, N.; Chen, G.; Wu, H.; Wang, Y.; Li, X.o.; Peng, L.; Xu, W.; Li, T.; Wang, Y.; Zhang, L.-F.; et al. H3.3-K27M neoantigen vaccine elicits anti-tumor T cell immunity against diffuse intrinsic pontine glioma: The phase I ENACTING trial. **2023**, *41*, 2052-2052, doi:10.1200/JCO.2023.41.16_suppl.2052.
31. Patel, N.; Kilburn, L.B.; Fonseca, A.; Stabingas, K.; Clanton, R.; Marcus, S.; Vezina, G.; Nazarian, J.; Keating, R.F.; Packer, R.J. Phase 1/2, first-in-child study of sonodynamic therapy (SDT) using low intensity focused ultrasound and 5-aminolevulinic acid (ALA) for patients with diffuse intrinsic pontine glioma. **2023**, *41*, TPS10070-TPS10070, doi:10.1200/JCO.2023.41.16_suppl.TPS10070.
32. McCrea, H.J.; Ivanidze, J.; O'Connor, A.; Hersh, E.H.; Boockvar, J.A.; Gobin, Y.P.; Knopman, J.; Greenfield, J.P. Intraarterial delivery of bevacizumab and cetuximab utilizing blood-brain barrier disruption in children with high-grade glioma and diffuse intrinsic pontine glioma: results of a phase I trial. *Journal of neurosurgery. Pediatrics* **2021**, *28*, 371-379, doi:10.3171/2021.3.Peds20738.
33. Abdelbaki, M.; Setty, B.; DeWire, M.D.; Cripe, T.P.; Curry, R. A pediatric and young adult phase I dose escalation study of BXQ-350 for solid and central nervous system tumors. **2020**, *38*, 2541-2541, doi:10.1200/JCO.2020.38.15_suppl.2541.
